# Quassinoids from Twigs of *Harrisonia perforata* (Blanco) Merr and Their Anti-Parkinson’s Disease Effect

**DOI:** 10.3390/ijms242216196

**Published:** 2023-11-11

**Authors:** Min Cai, Xiao-Lin Bai, Hao-Jing Zang, Xiao-Han Tang, Ying Yan, Jia-Jia Wan, Min-You Peng, Hong Liang, Lin Liu, Feng Guo, Pei-Ji Zhao, Xun Liao, Ying-Tong Di, Xiao-Jiang Hao

**Affiliations:** 1 State Key Laboratory of Phytochemistry and Plant Resources in West China, Kunming Institute of Botany, Chinese Academy of Sciences, Kunming 650201, China; caimin@mail.kib.ac.cn (M.C.); zanghaojing@mail.kib.ac.cn (H.-J.Z.); tangxh1063@gmail.com (X.-H.T.); crystal_yanying@126.com (Y.Y.); wanjiajia@mail.kib.ac.cn (J.-J.W.); pmy3419@126.com (M.-Y.P.); lianghong@mail.kib.ac.cn (H.L.); liulin@mail.kib.ac.cn (L.L.); guofeng@mail.kib.ac.cn (F.G.); haoxj@mail.kib.ac.cn (X.-J.H.); 2 School of Life Sciences, Yunnan University, Kunming 650091, China; pjzhao@ynu.edu.cn; 3 University of Chinese Academy of Sciences, Beijing 100049, China; baixl@cib.ac.cn; 4 Chengdu Institute of Biology, Chinese Academy of Sciences, Chengdu 610041, China; 5 State Key Laboratory of Functions and Applications of Medicinal Plants & College of Pharmacy, Guizhou Provincial Engineering Technology Research Center for Chemical Drug R&D, Guizhou Medical University, Guiyang 550014, China

**Keywords:** *Harrisonia perforata*, quassinoid, simarubaceae, neuroprotection, PC12 cells, *PINK1^B9^* flies

## Abstract

Six new C-20 and one new C-19 quassinoids, named perforalactones F-L (**1**–**7**), were isolated from twigs of *Harrisonia perforata*. Spectroscopic and X-ray crystallographic experiments were conducted to identify their structures. Through oxidative degradation of perforalactone B to perforaqussin A, the biogenetic process from C-25 quassinoid to C-20 via Baeyer–Villiger oxidation was proposed. Furthermore, the study evaluated the anti-Parkinson’s disease potential of these C-20 quassinoids for the first time on 6-OHDA-induced PC12 cells and a *Drosophila* Parkinson’s disease model of *PINK1^B9^*. Perforalactones G and I (**2** and **4**) showed a 10–15% increase in cell viability of the model cells at 50 μM, while compounds **2** and **4** (100 μM) significantly improved the climbing ability of *PINK1^B9^* flies and increased the dopamine level in the brains and ATP content in the thoraces of the flies.

## 1. Introduction

Quassinoids are a group of structurally diverse natural products found in Simaroubaceae family plants, with over 500 ones identified to date [1]. These compounds can be categorized into six groups: C-26, C-25, C-22, C-20, C-19, and C-18 types, based on their carbon backbone [2]. Although their biogenetic pathway is not yet fully understood, it is believed that they originate from tetracyclic tirucallane triterpene and undergo a series of oxidation, rearrangement, and recyclization steps [3]. Additionally, these quassinoids have been found to exhibit a wide range of biological and pharmacological effects, including anti-inflammatory, anti-tumor, and anti-malarial properties [1,3,4,5,6,7]. Therefore, this type of compounds has become an area of significant interest in the scientific community [8,9].

**Figure 1:**
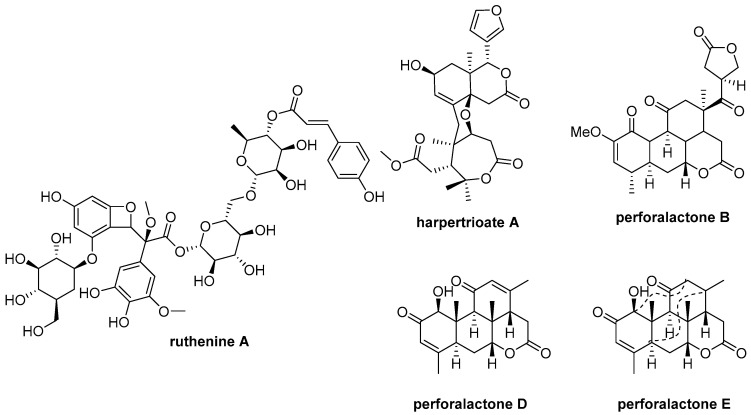
Chemical structures of ruthenine A, harpertrioate A, perforalactone B, perforalactones D and E.

Parkinson’s disease (PD) is a common neurodegenerative condition [10]. Unfortunately, there is currently no cure available, and the drugs that are available only provide symptomatic relief and can cause side effects [11]. As a result, there is a pressing need for new anti-PD drugs [12,13]. The PTEN-induced putative kinase 1 (PINK1) is a promising drug target [11], as mutations in these gene have been linked to early-onset PD [6,14,15]. Researchers have been exploring a variety of natural products for potential anti-PD compounds, and one such product is ruthenine A from black wolfberry (Figure 1) [16]. PINK1 loss-of-function flies (*PINK1^B9^*), a PD animal model, have exhibited PD-like symptoms, such as degeneration of flight muscle and dopaminergic neurons, shortened lifespan, and impaired locomotor abilities [17,18].

*Harrisonia perforata* (Bl.) Merr. belongs to the Simarubaceae family and is a shrub plant [19]. A number of chemical components have been identified in previous studies, including quassinoids, limonoids, chromones, and polyketides [4,6,20,21,22]. Our previous research found that some of these compounds exhibit biological activities against neurodegenerative disease through various mechanisms (Figure 1). For example, harpertrioate A reduced A*β*42 and A*β*40 production and shifted APP processing toward a nonamyloidogenic pathway, while perforalactones D and E significantly induced lysosomal biogenesis through transcriptional activation of lysosomal genes [6,22]. As part of our ongoing work to discover [23] bioactive molecules from plant [14,20,21,24,25,26,27], we isolated seven undescribed quassinoids (**1**–**7**), including six C-20 and one C-19 quassinoids from the twigs of this plant. We also verified the oxidative degradation of C-25 quassinoid, perforalactone B (Figure 1), to the C-20 quassinoid, perforaquassin A, through chemical transformation, and then the crucial “link” between C-25 type and C-20 type quassinoids was proposed. Additionally, these quassinoids were tested for their anti-PD potential using 6-OHDA-induced PC12 cells and the Drosophila Parkinson’s disease model of *PINK1^B9^*. The results showed promising bioactive properties, making these newly discovered quassinoids potential candidates for further development as anti-PD drugs.

## 2. Results

### 2.1. Structure Elucidation

**Figure 2:**
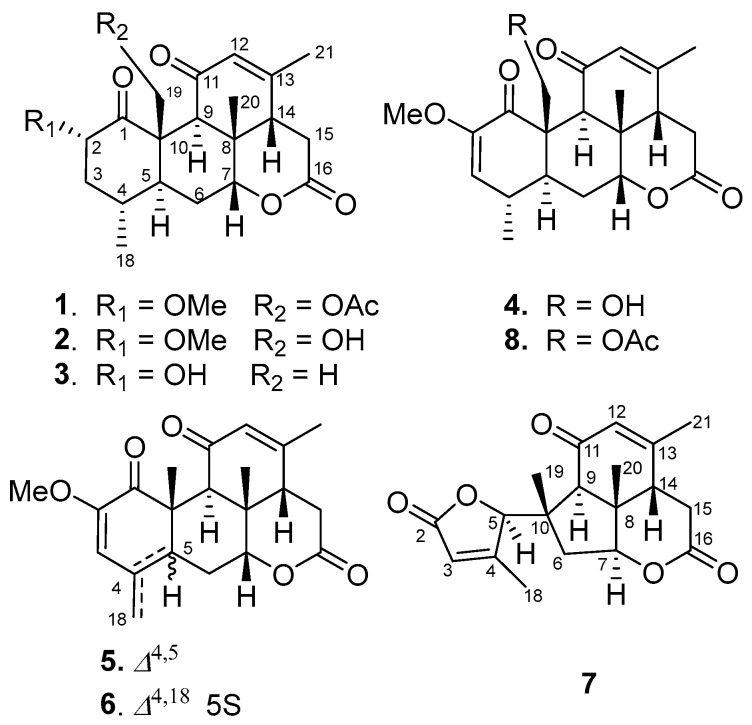
Structures of compounds **1**–**8**.

Seven undescribed quassinoids, perforalactones F-L (**1**–**7**), were isolated from the twigs of *H. perforata* plant using solvent partitioning and various types of chromatography techniques, including normal, reverse, and molecular-exclusion (Figure 2).

**Figure 3:**
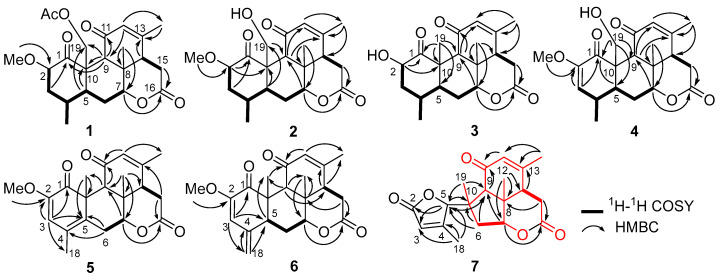
^1^H-^1^H COSY and key HMBC correlations of compounds **1**–**7**.

**Figure 4:**
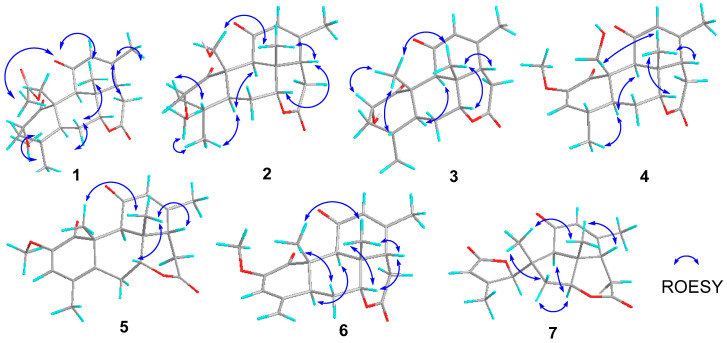
Key ROESY correlations of compounds **1**–**7**.

Perforalactone F (**1**) was obtained as a white powder. Its molecular formula C_23_H_30_O_7_ was established by the HRESIMS (*m*/*z* 441.1879 [M + Na]^+^, calculated for C_23_H_30_O_7_Na, 357.1516) with nine degrees of unsaturation. The IR spectrum showed absorption bands for *δ*-lactone (1731 cm^−1^) and *α*,*β*-unsaturated ketone (1675 cm^−1^). The ^13^C NMR spectrum of Perforalactone F (**1**) (Table 1, Figure S2) revealed 23 signals, which were further differentiated by the DEPT to be five methyls (one oxygenated), four methylenes (one oxygenated), seven methines (including one olefinic and two oxygenated), and seven non-hydrogenated carbons (including four carbonyls). The ^1^H NMR spectrum of perforalactone F (**1**) (Figure S1) showed signals due to one olefinic proton [*δ*_H_ 5.83 (1H, br d)], one secondary methyl [*δ*_H_ 0.96 (3H, d, *J* = 6.6 Hz)], three tertiary methyls [*δ*_H_ 1.25 (3H, s), 1.96 (3H, d, *J* = 1.2 Hz), and 2.12 (3H, s), and one methoxyl 3.46 (3H, s)] (Table 1). Its spectroscopic data are similar to those of C_20_ quassinoid, perforalactone C (**8**), except that the signals for the trisubstituted double bond at C-2/C-3 in **8** were replaced by those for 2,3-saturated single bond in perforalactone F (**1**) (Table 1) [4]. The assignment of the structure of perforalactone F (**1**) was confirmed by the ^1^H-^1^H COSY and HSQC correlations of C2-C3-C4(Me-18)-C5-C6-C7 (Figure 3 and Figures S3–S5). The H-2 (*δ*_H_ 4.60) was assigned as *β*-axial due to the large coupling constant (12.3, between H-2 and H-3a) and the presence of a strong ROE with H_2_-19 (Figure 4 and Figure S6) [4]. Consequently, the structure of perforalactone F (**1**) was assigned as shown.

The HRESIMS data [*m*/*z* 399.1767, (M + Na)^+^] suggested that the molecular formula of perforalactone G (**2**) is C_21_H_28_O_6_, with eight degrees of unsaturation. After comparing the 1D NMR data (Table 1, Figures S7 and S8) of perforalactone F (**1**) and perforalactone G (**2**), it was found that the absence of acetyl at C-19 in the latter is the only difference. The structural assignment of perforalactone G (**2**) was confirmed via 2D NMR spectra (Figures S9–S12), as illustrated in Figure 3 and Figure 4.

Perforalactone H (**3**) has a molecular formula of C_20_H_26_O_5_, which is 14 mass units less than that of perforalactone G (**2**), according to HRESIMS [*m*/*z* 347.1840 (M + H)^+^]. The NMR data of perforalactone H (**3**) are similar to those of perforalactone G (**2**) (Table 1, Figures S13 and S14), indicating their structural similarity. However, signals corresponding to tertiary methyl in the NMR spectra of perforalactone H (**3**) were observed, instead of the signals for hydroxymethyl at C-10 and *O*-bear methyl at C-2 in perforalactone G (**2**). This was further confirmed via 2D NMR experiments (Figures S15–S18), and its planar structure and relative configuration are shown in Figure 3 and Figure 4, respectively.

According to HRESIMS analysis, perforalactone I (**4**) has a molecular formula of C_21_H_26_O_6_ [*m*/*z* 379.1511 (M + Na)^+^ (calculated for C_21_H_24_O_5_Na, 379.1516)], which is 42 mass units less than perforalactone C (**8**). Further analysis of its NMR data (Table 2, Figures S19 and S20) revealed that its structure closely resembles that of the latter, except for the absence of acetyl at C-19. This was confirmed via the HMBC correlations of H_2_-19 to C-1, C-5, and C-9 (Figures S21–S24). Thus, the structure of perforalactone I (**4**) was depicted as shown in Figure 3 and Figure 4.

Perforalactone J (**5**) is a white amorphous powder with a molecular formula of C_21_H_24_O_5_, as determined by HRESIMS analysis (*m*/*z* 379.1511 [M + Na]^+^ (calculated for C_21_H_24_O_5_Na, 379.1516)). By comparing its 1D NMR data with those of perforalactone I (**4**) (Table 2, Figures S25 and S26), their close resemblance suggested that both compounds shared the same picrasane skeleton, except the following two facts: first, the CH(4)-CH(5) single bond in perforalactone I (**4**) was dehydrogenated to a double bond (*δ*_C_ 125.1, C-4; 133.7, C-5) in perforalactone J (**5**); second, the hydroxymethyl at C-10 in perforalactone I (**4**) was replaced by a methyl (*δ*_C_ 24.5, *δ*_H_ 1.63) in perforalactone J (**5**). Further 2D-NMR analyses verified these suggestions (Figures S27–S30). Thus, the structure of perforalactone J (**5**) was determined as shown (Figure 3 and Figure 4).

A white powder identified as perforalactone K (**6**) was found to have the same molecular formula, C_21_H_25_O_5_, as perforalactone J (**5**) according to HRESIMS [*m*/*z* 357.1704, (M + H)^+^]. However, the ^1^H and ^13^C NMR spectroscopic data of perforalactone K (**6**) (Table 2, Figures S31 and S32) showed the presence of exomethylene (*δ*_C_ 113.0, *δ*_H_ 4.94, 5.18) instead of sp^2^ quaternary carbon in perforalactone J (**5**). These data suggest that a C(4)-C(18) double bond in perforalactone K (**6**) replaced the C(4)-C(5) double bond in perforalactone J (**5**), which was confirmed by the HMBC correlations from H_2_-18 to C3, C-4, and C-5. The remaining part of the structure and the relative configuration of perforalactone K (**6**) was similar to that of perforalactone J (**5**), as determined via the HMBC and ROESY NMR spectra (Figures S3, S4 and S33–S36).

Compound **7**, identified as perforalactone L, was obtained as white needle crystals and displayed a peak at *m*/*z* 353.1365 [M + Na]^+^ (calculated for C_19_H_22_O_5_Na, 353.1359) in HRESIMS, indicating a molecular formula of C_19_H_22_O_5_ with nine degrees of unsaturation. Further analysis of the ^13^C NMR spectrum (Table 2, Figure S38) revealed 19 signals, including 4 methyls (one oxygenated), 2 methylenes, 6 methines (including 2 olefinic and 2 oxygenated), and 7 non-hydrogenated carbons (including 3 carbonyls and 2 olefinic). The ^1^H NMR spectrum displayed signals due to two olefinic protons [*δ*_H_ 5.88 (1H, dd, *J* = 3.0,1.5 Hz); *δ*_H_ 5.76 (1H, d, *J* = 1.5 Hz)] and four tertiary methyls [*δ*_H_ 1.16 (3H, s), 1.31 (3H, s), and 1.94 (3H, s), and 2.19 (3H, s)] (Table 2, Figure S37). Except for three carbonyls and two trisubstituted double bonds, the remaining four degrees of unsaturation indicated the presence of a four-ring system in perforalactone L (**7**). All the data suggested that perforalactone L (**7**) is a C-19 quassinoid.

**Figure 5:**
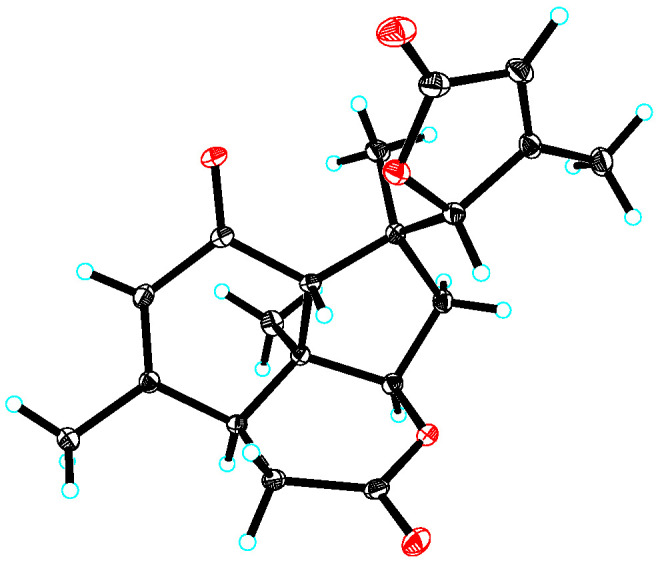
X-ray crystallographic structure of **7**.

According to 2D NMR experiments (^1^H-^1^H COSY, HSQC, and HMBC), it was discovered that perforalactone L (**7**) was made up of two components (as shown in Figures S3 and S39–S42). The HMBC correlations indicated that the right component (in red) contained three fused ring systems (two six-rings and one five-ring system) with *δ*-lactone, a double bond between C-12 and C-13, a ketone group at C-11, three methyls at C-8, 10, and 13, respectively, and a methylene at C-6, which was the same as that of perforalactone I (**4**). In the left component (in black), HMBC correlations of H_3_-18/C-3, C-4, and C-5, and of H-5/C-2 and C-3 suggested the presence of *γ*-lactone with methyl at C-4. Furthermore, HMBC correlations of H-6 and H_3_-19/C-5 and of H-5/C-9 and C-10 indicated that both components were joined by the bond C-5 and C-10. Thus, it was determined that perforalactone L (**7**) has a 1,2-*seco*-1-nor-6(5→10)-*abeo*-picrasan-2,5-olide skeleton. Subsequently, through an X-ray diffraction experiment (ccdc No. 2285369; flack parameter = 0.04(8)) (Table S1, Figure 5), it was unambiguously determined that the absolute configuration of **7** was 5*R*, 7*R*, 8*S*, 9*R*, 13*S*, and 14*S*.

### 2.2. Chemical Transformation from C_25_ Quassinoids to C_20_ and Their Proposed Biogenetic Pathway

**Scheme 1:**
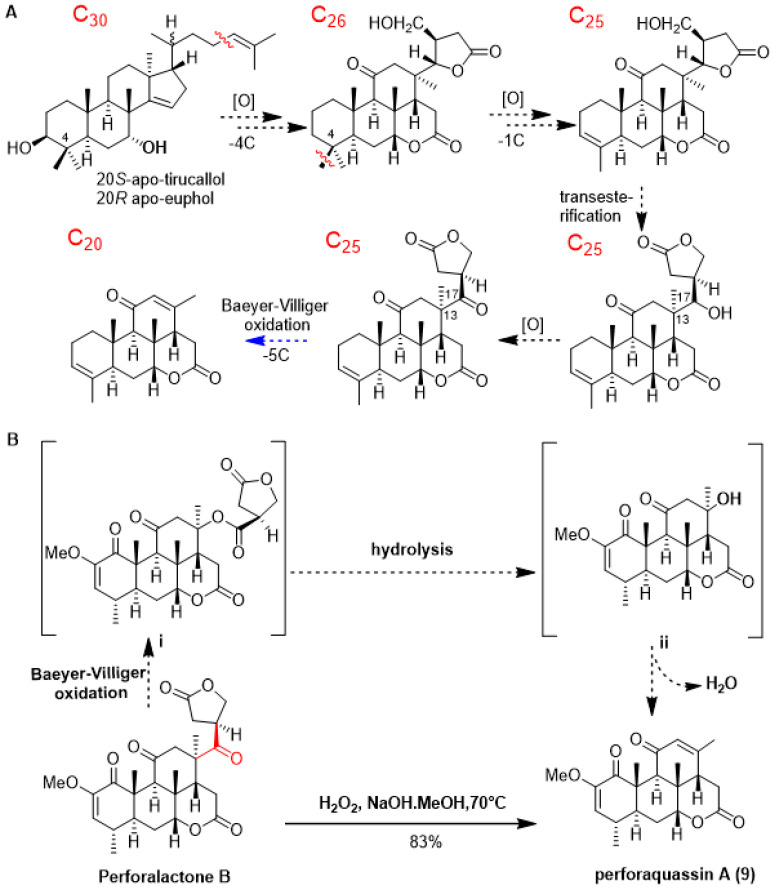
(**A**) Updated hypothesis for the biosynthetic pathway of C_20_ quassinoids, and proposed step in this work in blue. (**B**) Chemical transformation (in plain) and proposed mechanism (in dashed) of quassinoid from C_25_ quassinoid to C_20_ one.

As shown in Scheme 1A, quassinoids are known to be derived from tetracyclic triterpene precursors through a series of oxidative degradation processes. To date, several biogenetic intermediates have been found suggesting how C_30_ triterpenoids are degraded to C_20_ quassinoids; however, no chemical transformation was accomplished [1]. Since C_25_ quassinoid perforalactone B structurally resembles the C_20_ quassinoids perforalactones F-L, along with the fact that both types of quassinoids coexist in *H. perforata*, we surmised that Baeyer–Villiger oxidation would be a straightforward gateway to the fission of the C-13/C-17 bond in perforalactone B [28]. After trying a number of reaction conditions, we found that perforalactone B in the presence of H_2_O_2_ and NaOH in MeOH at 70 °C overnight could be converted to a product with 83% yield (Table S2). The product displayed a peak at *m*/*z* 359 [M + H]^+^. Further analysis of its ^13^C NMR spectra (Table 2) revealed 21 signals, including 5 methyls (1 oxygenated), 2 methylenes, 6 methines (including 2 olefinic and 1 oxygenated), and 7 non-hydrogenated carbons (including 3 carbonyls and 2 olefinic). Its structure was identified to be a C_20_ quassinoid, perforaquassin A (**9**), by comparison of the NMR data with the literature [29]. On the basis of these results and the literature precedent, we propose a plausible route for this reaction, as shown in Scheme 1B. Perforalactone B could be further transformed to a key intermediate **i** via a mechanism similar to that of chemical Baeyer–Villiger oxidation. The insertion of an oxygen atom could occur between C-13 and C-17 because the fully substituted C-13 carbon has a migratory aptitude comparable to that of the *γ*-lactone group. The intermediate **i** would then undergo hydrolysis/dehydration to produce perforaquassin A (**9**). Thus, chemical transformation from perforalactone B to perforaquassin A (**9**) provides new insight into understanding the oxidative degradation pathway of quassinoids.

### 2.3. Neuroprotective Activity of the Isolated Compounds on 6-OHDA-Induced Injury of PC12 Cells

**Figure 6:**
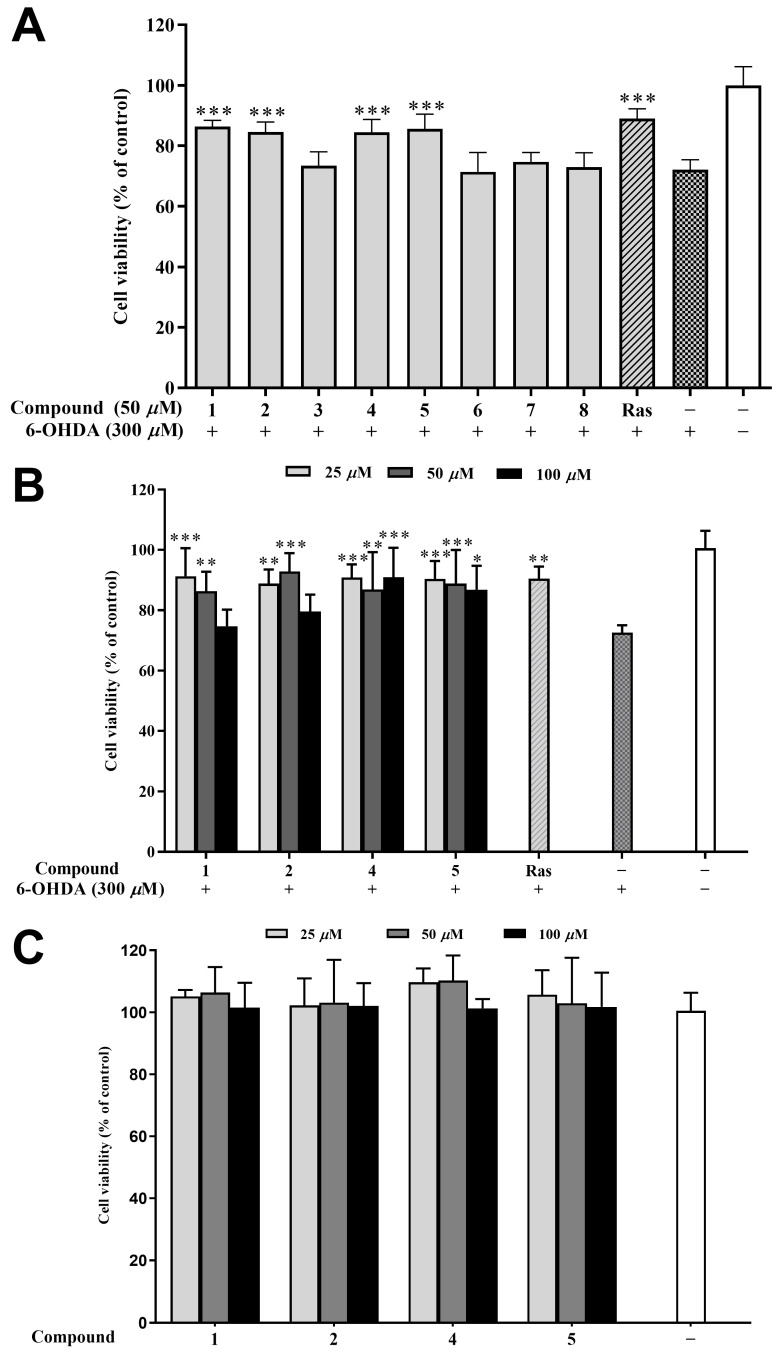
Neuroprotective effects of isolated compounds against 6-OHDA-induced injury in PC12 cells. (**A**) Neuroprotective effect of the isolated compounds at 50 μM. (**B**) Neuroprotective effect of compounds **1**, **2**, **4**, and **5** at various concentrations (25, 50, and 100 μM). (**C**) Cytotoxic effect of compounds **1**, **2**, **4**, and **5** (25, 50, and 100 μM) on the PC12 cell. Data were expressed as the means ± SD of three independent experiments. * *p* < 0.05, ** *p* < 0.01, and *** *p* < 0.001 compared with model cells in (**A**,**B**) and compared with control cells in (**C**). Rasagiline (Ras) was used as a positive control.

6-Hydroxydopamine (6-OHDA) is an oxidative neurotoxin and a redox cycling dopamine analog that was used to generate Parkinson’s disease (PD) models in cells and animals. Based on our previous report, a dose of 6-OHDA was determined for the C12 cells. The main mechanism of the neurotoxicity of 6-OHDA includes the induction of oxidative stress [30], while rasagiline has a neuroprotective effect *in vitro* in PC12 cells against a number of toxins such as 6-hydroxydopamine (6-OHDA), MPTP, etc. [31]. Therefore, rasagiline has been usually used as the positive control in this model for screening compounds of anti-PD potential [32,33]. In the preliminary experiment testing, four compounds (**1**, **2**, **4**, and **5**) at 50 μM improved the cell viability of 6-OHDA-induced PC12 cells by 10–15% compared to the model group (Figure 6A). Further, different concentrations (25, 50, and 100 μM) of **1**, **2**, **4**, and **5** were tested, and all the samples improved the cell viability except for compounds **1** and **2** at 100 μM (Figure 6B). It is worth noting that 50 μM of compound **2** exhibited the strongest cytoprotective effect (92.84% of cell viability), surpassing even the positive control rasagiline (90.48%). Furthermore, the cytotoxicity of compounds **1**, **2**, **4**, and **5** at different concentrations (25, 50, and 100 μM) is extremely low, as demonstrated in Figure 6C. This study is the first report on the neuroprotective effects of compounds from *H. perforata*, providing a fresh perspective for the development and design of anti-PD drugs [34].

### 2.4. Effect of the Isolated Compounds on Locomotor Ability of PINK1^B9^ Flies

**Figure 7:**
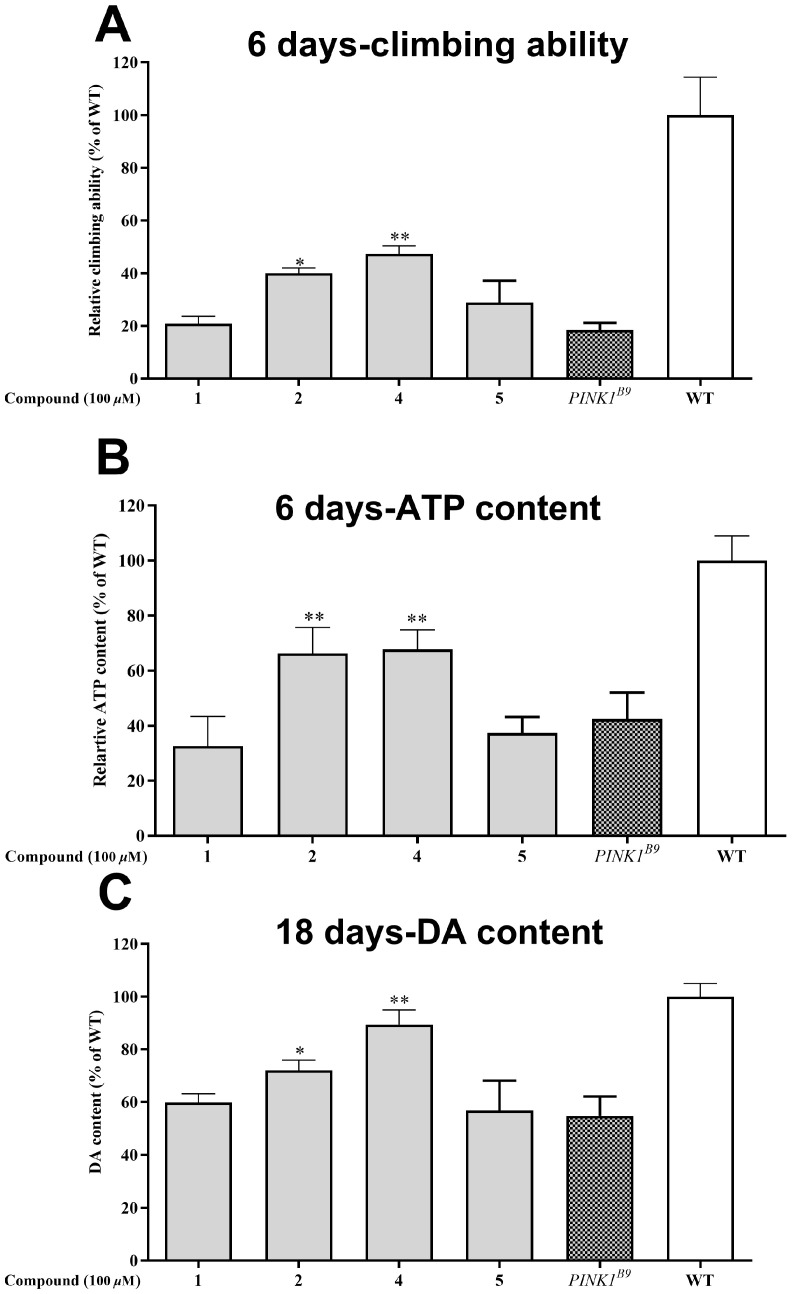
Neuroprotective effect of the isolated compounds (**1**, **2**, **4**, and **5** at the concentration of 0.1% (*v*/*v*)) on *PINK1^B9^* flies. (**A**) Climbing abilities of WT flies, *PINK1^B9^*, and *PINK1^B9^* treated with isolated compounds at 6 days. (**B**) ATP levels of WT flies, *PINK1^B9^*, and *PINK1^B9^* treated with isolated compounds at 6 days. (**C**) Dopamine levels of WT flies, *PINK1^B9^*, and *PINK1^B9^* treated with isolated compounds at 18 days. Data from three times independent experiments were expressed as means ± SD, and * *p* < 0.05, and ** *p* < 0.01 compared with *PINK1^B9^* flies.

Out of the seven compounds tested, **1**, **2**, **4**, and **5** displayed superior neuroprotective effects in cell experiments. Consequently, we proceeded to investigate their impact on the *Drosophila* PD model of *PINK1^B9^*. Following a six-day diet containing 100 μM of the compounds, we evaluated the locomotor ability through a climbing experiment. Figure 7A shows that the *PINK1^B9^* flies’ climbing ability was only 18.46% that of the WT flies. However, administering compounds **2** and **4** significantly improved their performance to 40.01 and 47.33%, respectively.

### 2.5. Effect of the Isolated Compounds on ATP Contents of PINK1^B9^ Flies

The role of PINK1 in preserving mitochondrial homeostasis cannot be overstated; when it is absent, ATP production is significantly reduced. ATP levels are a widely accepted measure of mitochondrial function, and in this study, the thoracic ATP content of the *PINK1^B9^* flies was only 42.41% that of the WT flies at 6 days of age (as depicted in Figure 7B). However, the administration of compounds **2** and **4** (at a concentration of 100 μM) led to a substantial increase in ATP content for the *PINK1^B9^* flies, with levels reaching 66.26 and 67.70%, respectively, at this age.

### 2.6. Effect of the Isolated Compounds on Dopamine Content of PINK1^B9^ Flies

A decrease in dopamine levels is a typical characteristic of Parkinson’s disease patients and *PINK1^B9^* flies. Figure 7C illustrates that the dopamine content in *PINK1^B9^* flies was notably lower (54.8%) than that of WT flies at 18 days. However, administering compounds **2** and **4** (100 μM) resulted in a significant increase in dopamine levels, with relative levels reaching 71.99% and 89.45% at 18 days, respectively.

## 3. Discussion

While past reports focused mainly on the antifeedant effect of quassinoids, [4] we found, for the first time, the anti-PD potential of these compounds. The toxicity of 6-OHDA in PC12 cells has been shown to be directly related to its auto-oxidation and induction of oxidative stress, thus causing mitochondrial dysfunction and cell apoptosis [35]. Recently, a new alkaloid named chaetonigrisin G, isolated from the fungus *Chaetomium nigricolor* YT-2, showed significant neuroprotective activity through the upregulation of anti-oxidative (*SOD1*) and anti-apoptosis gene (*Bcl-2*) expression [36]. The new quassinoids (**1**, **2**, **4**, and **5**) in this work showed an equal or better effect on the 6-OHDA-induced PC12 cells compared to chaetonigrisin G. This suggests that these new quassinoids (**1**, **2**, **4**, and **5**) might also exert neuroprotective effects through anti-oxidative and anti-apoptosis mechanisms.

PINK1 is a mitochondria-localized serine-threonine kinase which plays important roles in combating oxidative stress and mitochondrial dysfunction. The *PINK1*-knock-out fruit flies (*PINK1^B9^* flies) exhibit PD-like phenotypes including impaired motor ability, reduction of dopamine and ATP levels, shortened lifespan, and degeneration of dopaminergic neurons [18]. Thus, *PINK^B9^* flies are an excellent PD animal model in the discovery of anti-PD drugs. Recently, ginseng protein was found to delay the PD-like phenotype of *PINK^B9^* flies, and up-regulate the key markers (Hsp60, mtHsp70, and CG5045) of the UPR^mt^ pathway to maintain the mitochondrial homeostasis [37]. Another study reported that grape skin extract exhibited the likely anti-PD activity in *PINK^B9^* flies and enhanced the mitochondrial autophagy pathway by upregulating the LC3-II/LC3-I ratio and reducing p62 accumulation [38]. Compared to previous studies, the new quassinoids (**2** and **3**) improved the motor ability in *PINK1^B9^* flies more than grape skin extract and increased the level of ATP in *PINK1^B9^* flies to a much greater extent than ginseng protein. Therefore, we speculate that these two new quassinoids might regulate the anti-oxidative and mitochondrial autophagy-related pathway.

Overall, seven new quassinoids (**1**–**7**) were discovered in the twigs of *H. perforata* during this study. The structural diversity of these quassinoids is extensive and contributes to our understanding of *H. perforata*’s chemical composition. This research also revealed the transformation of C-25-type quassinoids into C-20-type quassinoids via chemical means, a first-time demonstration. Additionally, we discovered the remarkable anti-PD potential in C-20 quassinoids, another first. This work lays the groundwork for further investigation into *H. perforata*’s properties and provides a potential avenue for developing Parkinson’s disease medication.

## 4. Materials and Methods

### 4.1. General Experimental Procedures

^1^H and ^13^C NMR were measured by DRX-500, Avance III-600, 800 MHz superconducting NMR (Bruker, Zurich, Switzerland) with TMS as the internal standard; ESIMS and HREIMS data were recorded on an Agilent 1290 UPLC/6540 Q-TOF high-performance liquid chromatography/quadrupole time-of-flight mass spectrometer (Agilent, Redwood City, CA, USA); and infrared spectra were recorded on a Bruker Tensor-27 instrument using KBr pellets. HPLC separation was performed on an Agilent Infinity 1260 liquid chromatograph (Agilent Technologies, Waldbronn, Germany) with XSelect CSH C-18 analytic (4.6 × 150 mm) and semi-Prep (9.8 × 150 mm) column (all are 5 μm, Waters, MA, USA). TLC was performed on precoated TLC plates (200–250 μm thickness, F254 Si gel 60, Qingdao Marine Chemical, Inc., Qingdao, China) with compounds visualized by spraying the dried plates with 10% aqueous H_2_SO_4_ followed by heating until dryness.

### 4.2. Plant Material, Extraction, and Isolation

The plant source was the twigs of *H. perforata* from Hainan Province, China, obtained in January 2018. The samples were collected and identified by Dr. Shengzhuo Huang, assistant professor at Hainan Institute of Tropical Biotechnology. The specimens (NO. 20180104) are kept in the State Key Laboratory of Phytochemistry and Sustainable Utilization of Western Plant Resources, Kunming Institute of Botany, Chinese Academy of Sciences.

The powder of *H. perforate* twigs (200–300 mesh, 100 kg) was extracted with methanol three times. The combined methanol extracts were evaporated under reduced pressure; then, the obtained residue was suspended in H_2_O and partitioned with petroleum ether (PE) and ethyl acetate (EtOAc) successively. The EtOAc partition fraction (1.0 kg) was fractionated into five fractions (Fr. 1–5) using MCI gel CC elution with gradient methanol-H_2_O (3:7–1:0). Then, fr. 3 (45.7 g) was further subjected to silica gel CC to yield nine fractions (Fr. 3.1–3.9) with gradient PE–EtOAc (10:1–0:1) and MeOH. Fr. 3.3 was further purified by Sephadex LH-20 (CH_2_Cl_2_–MeOH), and we repeated semipreparative HPLC (MeCN–H_2_O; 30: 70–55: 45) separations to yield **1** (1.1 mg), **2** (0.9 mg), **3** (6 mg), **4** (72 mg), **5** (1.2 mg), and **6** (11.7 mg). The Fr. 3.2 was subjected to Sephadex LH-20 (CH_2_Cl_2_–MeOH) and semipreparative HPLC (MeCN–H_2_O; 54: 46) to obtain **7** (5.9 mg).

### 4.3. Compound Characterization

Perforalactone F (**1**): white amorphous powder; [α]^23^_D_ −64.8 (*c* 0.08, MeOH); IR (KBr) *ν*_max_ 3435, 2959, 2927, 2856, 1731, 1675, 1637, 1657, 1462, 1442, 1383, 1275, 1231, 1167, 1124, and 1038 cm^−1^; ESI-MS(+) *m*/*z* 418 [M + Na]^+^; HR-ESI-MS(+) *m*/*z* 441.1879 [M + Na]^+^ (calculated for C_23_H_30_O_7_Na, 441.1884). ^1^H and ^13^C NMR data are shown in Table 1.

Perforalactone G (**2**): white amorphous powder; [α]^23^_D_ −46.2 (*c* 0.06, MeOH); IR (KBr) *ν*_max_ 3439, 2963, 2927, 2873, 2856, 1723, 1671, 1636, 1440, 1383, 1275, 1251, 1229, 1059, 992, 978, 890, and 811 cm^−1^; ESI-MS(+) *m*/*z* 347 [M + H]^+^; HR-ESI-MS(+) *m*/*z* 347.1840 [M + H]^+^ (calculated for C_20_H_27_O_5_, 347.1853). ^1^H and ^13^C NMR data are shown in Table 1.

Perforalactone H (**3**): white amorphous powder; [α]^23^_D_ −93.2 (*c* 0.05, MeOH); IR (KBr) *ν*_max_ 3424, 2961, 2924, 2853, 1725,1661,1638, 1469, 1440, 1383, 1315, 1256, 1226, 1110, 1037, 985, 970, 864, and 810 cm^−1^; ESI-MS(+) *m*/*z* 399 [M + Na]^+^; HR-ESI-MS(+) *m*/*z* 399.1767 [M + Na]^+^ (calculated for C_21_H_28_O_6_Na, 399.1778). ^1^H and ^13^C NMR data are shown in Table 1.

Perforalactone I (**4**): white amorphous powder; [α]^23^_D_ +88.5 (*c* 0.05, MeOH); IR (KBr) *ν*_max_ 3428, 2960, 2925, 2876, 2852, 1732, 1670, 1664, 1638, 1461, 1440, 1383, 1250, 1114, 1080, 1040, 939, 868, and 860 cm^−1^; ESI-MS(+) *m*/*z* 375 [M + H]^+^; HR-ESI-MS(+) *m*/*z* 375.1794 [M + H]^+^ (calculated for C_21_H_27_O_6_, 375.1802). ^1^H and ^13^C NMR data are shown in Table 2.

Perforalactone J (**5**): white amorphous powder; [α]^23^_D_ −44.6 (*c* 0.08, MeOH); IR (KBr) *ν*_max_ 3435, 2964, 2926, 2853, 1743, 1671, 1647, 1441, 1384, 1301, 1279, 1263, 1230, 1196, 1114, 1072, 1053, 952, and 860 cm^−1^; ESI-MS(+) *m*/*z* 379 [M + H]^+^; HR-ESI-MS(+) *m*/*z* 379.1511 [M + Na]^+^ (calculated. for C_21_H_24_O_5_Na, 379.1516). ^1^H and ^13^C NMR data are shown in Table 2.

Perforalactone K (**6**): white amorphous powder; [α]^23^_D_ +171.0 (*c* 0.09, MeOH); IR (KBr) *ν*_max_ 3434, 3096, 2934, 2853, 1733, 1699, 1678, 1638, 1622, 1599, 1439, 1383, 1247, 1234, 1218, 1171, 1044, 977, 930, and 892 cm^−1^; ESI-MS(+) *m*/*z* 357 [M + Na]^+^; HR-ESI-MS(+) *m*/*z* 357.1704 [M + H]^+^ (calculated for C_21_H_25_O_5_, 357.1697). ^1^H and ^13^C NMR data are shown in Table 2.

Perforalactone L (**7**): white amorphous powder; [α]^23^_D_ −9.2 (*c* 0.07, MeOH); IR (KBr) *ν*_max_ 3411, 2974, 2918, 2850, 1754, 1743, 1684, 1635, 1576, 1446, 1415, 1436, 1326, 1202, 1046, and 986 cm^−1^; ESI-MS(+) *m*/*z* 353 [M + Na]^+^; HR-ESI-MS(+) *m*/*z* 353.1365 [M + Na]^+^ (calculated for C_19_H_22_O_5_Na, 353.1359). ^1^H and ^13^C NMR data are shown in Table 2. 

Perforaquassin A (**9**): ^1^H NMR (400 MHz, CDCl_3_) *δ*_H_ 5.84 (s, 1H), 5.29 (d, *J* = 2.2 Hz, 1H), 4.30 (d, *J* = 1.7 Hz, 1H), 3.58 (s, 3H), 2.94 (dd, *J* = 13.1, 5.4 Hz, 3H), 2.60 (dd, *J* = 18.7, 12.2 Hz, 2H), 2.46 (d, *J* = 6.6 Hz, 1H), 2.34 (dd, *J* = 12.1, 7.0 Hz, 1H), 2.09 (dd, *J* = 11.4, 3.2 Hz, 1H), 1.93 (s, 4H), 1.85 (d, *J* = 5.7 Hz, 2H), 1.58 (s, 2H), 1.53 (s, 4H), 1.18 (s, 4H), 1.11 (d, *J* = 6.9 Hz, 4H). ^13^C NMR (125 MHz, CDCl3) *δ*_C_ 197.8, 194.8, 169.0, 155.1, 169.1, 148.1, 127.1, 116.1, 82.2, 76.8, 55.1, 47.3, 45.6, 45.3, 43.5, 37.2, 31.3, 25.8, 22.4, 19.5, 12.8. MS (ESI+) [M ^+^ H]^+^: 359, found: 359.

### 4.4. X-ray Diffraction Analysis

X-ray Crystallographic Analyses of 7. Perforalactone L were crystallized from a mixed solvent system (acetone/H_2_O, 20:1) at rt. The X-ray crystallographic data for 7 have already been retrieved at the Cambridge Crystallographic Data Centre, CCDC number 2285369.

### 4.5. Cell Culture and Cell Viability Assays

The CCK-8 assay was used to determine the neuroprotective effect of the isolated compounds, as described previously [17]. The PC12 cells were cultured in Dulbecco’s Modified Eagle’s medium (DMEM, TransGen Biotech, FS101-02) with 10% (*v*/*v*) of fetal bovine serum (FBS) and 1% (*v*/*v*) of penicillin/streptomycin at 37 °C in 5% CO_2_ in a humid atmosphere. First, the test compound was dissolved in dimethyl sulfoxide (DMSO) and diluted with DMEM to a concentration of 50 μM. PC12 cells were seeded in 96-well microplates at a density of 10^4^ cells/well for 24 h. Second, the cells were pre-treated with the test compounds at 37 °C for 2 h, followed by cultivation with 300 μM of 6-OHDA for 24h. Finally, 10 μL of CCK-8 was added and incubated for 1 h before the UV absorbance was measured by a multimode microplate reader (Thermo Fisher, Waltham, MA, USA) at 450 nm.

### 4.6. Effects of the Isolated Compounds on PINK1^B9^ Flies

#### 4.6.1. Fly Strain and Husbandry

W^1118^ (WT) and *PINK1^B9^* [w (*) Pink1 (B9)] mutants were generously presented by Prof. Zhuohua Zhang in Xiangya School of Medicine, Central South University of China. The flies were maintained in an incubator with a regular fly medium at 25 ± 1 °C, 60% relative humidity, and a 12 h light/dark cycle. The regular fly medium was prepared using a mixture of 90 g yeast, 240 g corn, 32 g agar, 189 g glucose, 98 g sucrose, and 25 mL preservative per 3.5 L boiling water. Male flies of both *PINK1^B9^* and WT were collected for the following experiments. Experimental diets were prepared by adding 100 μM test compounds in Formula 4–24 Instant *Drosophila* Medium (Carolina Biological Supply Company, Burlington, NC, USA), which was changed every 2–3 days. The flies were divided into three groups: group Ι, WT flies fed with an unmedicated diet; group Ⅱ, *PINK1^B9^* flies fed with unmedicated food; and group Ⅲ, *PINK1^B9^* flies fed with the test compounds (100 μM).

#### 4.6.2. Climbing Assay

The locomotor ability of the flies was evaluated using an infrared behavioral recorder (Yihong Technology Co., Ltd., Wuhan, China). Cohorts of 20 flies (6 days old) from each group were subjected to the assay. Flies were first anesthetized using CO_2_, transferred into a vertical glass tube (15 cm in length and 1.5cm in diameter), and then acclimated at room temperature for 30 min. The glass tube was inserted into the recorder, and the locomotor ability of the flies was recorded every 5 min for at least 4 h at the same time of day.

#### 4.6.3. ATP Measurement

The contents of ATP in the thoraces of flies were measured by a luciferin–luciferase system using the ATP Assay Kit (Biyuntian Co., Ltd., Shanghai, China). Protein content was determined using the BCA method (BCA protein assay, Elabscience, Wuhan, China). Thoraces of five 6-day-old flies were collected and homogenized in the lysis buffer, which was centrifugated at 12,000× *g* at 4 °C for 5 min. The supernatant was mixed with a luminescent solution and detected by a Luminometer (Molecular Devices, San Jose, CA, USA) [18]. ATP content was calculated as a percentage of total protein for each sample. Relative ATP contents were determined, and experiments were repeated at least three times.

#### 4.6.4. Dopamine Measurement

The dopamine content in the flies’ brains was determined via a Dopamine Research ELISA kit (Enzyme Link Biotechnology Co., Ltd., Dongguan, China). Protein content was determined via the same method as in the above ATP measurement section. First, flies (30 flies in each group) were anesthetized and transferred into EP tubes which were frozen in liquid nitrogen. Second, the flies were decapitated, and the collected heads were homogenized in 50 μL of ice-chilled citrate acetate buffer (50 mM pH = 6.5) and then centrifugated at 12,000× *g* at 4 °C for 15 min. Finally, the supernatant was used to determine the dopamine level according to the manufacturer’s instructions. Optical density was measured at 450 nm by a multimode microplate reader (Thermo Fisher, USA). Dopamine content was calculated as a percentage of total protein for each sample. 

#### 4.6.5. Data Analysis

Each experiment was repeated at least three times. Statistical significance was measured using one-way analysis of variance (ANOVA) analyses with Dunnett’s multiple comparisons test. The significance is indicated as follows: * *p* < 0.05, ** *p* < 0.01, and *** *p* < 0.001. All statistics analyses were performed by GraphPad Prism 7 (GraphPad Software, San Diego, CA, USA).

## Figures and Tables

**Table 1:** ^1^H and ^13^C NMR spectral data of compounds **1**–**3**.

No.	1 ^a^		2 ^b^		3 ^a^	
	δ_C_, Type	δ_H_ (*J*, Hz)	δ_C_, Type	δ_H_ (*J*, Hz)	δ_C_, Type	δ_H_ (*J*, Hz)
1	208.8, C		213.5, C		207.2, C	
2	82.1, CH	5.17, dd (12.7, 1.7)	69.9, CH	4.80–4.91, m	81.9, CH	4.60, dd (12.3, 7.0)
3	43.3, CH_2_	α: 1.31, d (12.7) β: 2.33, ddd (12.7, 6.6, 4.1)	46.5, CH_2_	α: 1.14, d (12.1) β: 2.04, m	44.4, CH_2_	α: 1.34, dd (12.3, 12.3) β: 2.35, m
4	29.4, CH	2.17, m	28.4, CH	1.85, m	29.2, CH	2.08, m
5	46.1, CH	1.38, m	47.5, CH	1.79–1.92, m	47.4, CH	1.50, m
6	27.0, CH_2_	α: 2.17, m β: 1.58, m	26.4, CH_2_	α: 2.48, ddd (12.4,6.8.4.0) β: 2.36, dd (12.4, 6.8)	27.3, CH_2_	2.04, m 1.82, m
7	82.2, CH	4.58, dd (12.9, 6.6)	82.3, CH	4.31, t (2.7)	82.1, CH	4.32, t (2.7)
8	37.2, C		37.2, C		37.2, C	
9	48.4, CH	3.40, s	51.0, CH	3.20, s	47.1, CH	3.30, s
10	51.1, C		47.7, C		52.6, C	
11	200.2, C		194.9, C		194.1, C	
12	126.7, CH	5.94, d (1.20	126.6, CH	5.85, d (1.2)	126.6, CH	5.83, d (1.2)
13	158.3, C		157.2, C		156.8, C	
14	48.5, CH	2.43, dd (12.7, 6.5)	47.8, CH	2.02, dd (6.7, 12.4)	48, CH	2.40, dd (12.5, 6.6)
15	30.7, CH_2_	α: 2.64, dd (18.6, 12.7) β: 2.95, dd (18.6, 6.5)	31.2, CH_2_	α: 2.60, dd (18.7, 12.4) β: 2.96, dd (18.7, 6.7)	31.2, CH_2_	α: 2.61, dd (18.7, 12.5) β: 2.95, dd (18.7, 6.6)
16	168.4, C		169.0, C		168.7, C	
18	18.9, CH_3_	0.99, d (6.4)	18.4, CH_3_	0.93, d (6.5)	18.9, CH_3_	0.96, d (6.6)
19	60.8, CH_2_	a: 4.12, d (12.6) b: 4.06, d (12.6)	15.2, CH_2_	1.44, s	62.3, CH_2_	a: 4.74, d (13.0) b: 4.46, d (13.0)
19-OAc					170.5, CH_2_	
19-OAc(Me)					21.4, CH_3_	2.12, s
19-OH		2.01, s				
20	23.8, CH_3_	1.23, s	23.1, CH_3_	1.19, s	23, CH_3_	1.25, s
21	22.4, CH_3_	2.00, d (1.0)	22.4, CH_3_	1.96, d (1.0)	22.5, CH_3_	1.94, d (1.2)
OMe	58.0, CH_3_	3.43, s			58.1, CH_3_	3.46, s

^a^: 800 MHz, CDCl_3_; ^b^:600 MHz, CDCl_3_.

**Table 2:** ^1^H and ^13^C NMR spectral data of compounds **4**–**7**.

No.	4 ^c^		5 ^d^		6 ^c^		7 ^c^	
	*δ*_C_, Type	*δ*_H_ (*J* in Hz)	*δ*_C_, Type	*δ*_H_ (*J* in Hz)	*δ*_C_, Type	*δ*_H_ (*J* in Hz)	*δ*_C_, Type	*δ*_H_ (*J* in Hz)
1	199.5, C		202.1, C		196.9, C			
2	157.1, C	4.60, dd (12.3, 7.0)	158.3, C		155.6, C		194.9, CH	
3	150.5, CH		133.7, CH	6.15, s	114.2, C	6.07, s	120.5, C	5.88, dd (3.0, 1.5)
4	41.2, CH	4.31, dd (3.5, 1.4)	150.7, C		149.3, CH		166.5, CH	
5	38.0, CH	1.81, ddd (4.5, 9.4, 2.1)	117.3, C		50.9, CH	2.11, d (2.8)	91.3, CH_2_	4,68, s
6	26.8, CH_2_	α: 2.09, dt (14.5, 3.5) β: 1.67, dd (14.5, 2.1)	33.5, CH_2_	α: 3.24, dd (15.5, 6.7) β: 2.58, d (15.5)	40.6, CH_2_	α: 2.85, m β: 2.09, t (2.8)	44.3, CH	α: 2.39, d (15.6) β: 2.15, dd (15.6, 5.0)
7	60.9, CH	4.06, t (11.8)	82.5, CH	4.46, t (2.8)	82.0	4.38, t (2.8)	86.9, C	4.62, d (5.0)
8	30.8, C		28.3, C		37.6, C		22.1, CH	
9	32.4, CH	3.24, s	39.5, CH	3.35, s	45.2, CH	3.04, s	52.1, C	2.91, s
10	47.8, C		49.3, C		46.3, C		44.8, C	
11	195.5, C		198.8, C		195.0, C		172.0, CH	
12	115.9, CH	5.39, d (2.7)	125.1, CH	5.82, s	127.0, CH	5.84, s	127.7, C	5.77, d (1.3)
13	126.9, C		127.2, C		141.5, C		157.0, CH	
14	47.4, CH	2.41, dd (12.3, 6.8)	49.4, CH	2.97, dd (16.9, 2.0)	47.3, CH	2.37, dd (12.0, 7.2)	45.1, CH_2_	2.42–2.49, m
15	52.4, CH_2_	α: 2.62, dd (18.6, 12.3) β: 2.96, dd (18.6, 6.8)	28.2, CH_2_	α: 2.70, dd (16.9, 2.0) β: 3.00, dd (16.9, 2.0)	31.1, CH_2_	α: 2.61, dd (18.8, 12.0) β: 2.97, dd 18.8, 7.2)	30.1, CH	α: 2.65, dd (18.4, 7.9) β: 2.97, dd (18.4, 7.9)
16	168.5, C		174.3, C		169.0, C		168.4, C	
18	20.4, CH_3_	1.16, d (6.8)	55.8, CH_3_	1.24, s	113.2, CH_2_	α: 5.18, d (1.7) β: 4.95, d (1.7)	44.5, CH_3_	2.19, s
19	81.8, CH_2_	a: 5.07, dd (11.9, 2.4) b: 3.93, dd (11.9, 2.4)	18.3, CH_3_	1.92, s	13.6, CH_3_	1.18, s	20.0, CH_3_	
19-OH		1.67, s						
20	23.0, CH_3_	1.21, s	24.7, CH_3_	1.64, s	25.6, CH_3_	1.38, s	16.5, CH_3_	1.31, s
21	22.2, CH_3_	1.98, d (1.2)	21.4, CH_3_	1.96, d (1.7)	22.3, CH_3_	1.93, s	21.2, CH_3_	1.94, s
OMe	55.5, CH_3_	3.62, s	62.3, CH_3_	3.74, s	55.5, CH_3_	3.36, s		

^c^: 500 MHz, CDCl_3_; ^d^: 800 MHz, CD_3_OD.

## Data Availability

The data underlying this study are available in the published article and its online supporting information.

## Supplementary Materials

The following supporting information can be downloaded at https://www.mdpi.com/article/10.3390/ijms242216196/s1.
