# Supplementary material for: Quassinoids from Twigs of Harrisonia perforata (Blanco) Merr and Their Anti-Parkinson’s Disease Effect
Source: Int J Mol Sci. 2023 Nov 11;24(22):16196. doi: 10.3390/ijms242216196 (PMC10671724; doi:10.3390/ijms242216196)
Supplement: Supplementary file 1 [file ijms-24-16196-s001.zip › supporting information-1019.pdf]

SUPPORTING INFORMATION

Quassinoids from Twigs of *Harrisonia Perforata* (Blanco) Merr and Their Anti-Parkinson's Disease Effect

Min Cai [1,2,3,†], Xiao-Lin Bai [3,4,†], Hao-Jing Zang [1,2,3], Xiao-Han Tang [1,2,3], Ying Yan [1,5], Jia-Jia Wan [1,3], Min-You Peng [1,5], Hong Liang [1,3], Lin Liu [1,3], Feng Guo [1,3], Pei-Ji Zhao [2], Xun Liao [4,*], Ying-Tong Di [1,*] and Xiao-Jiang Hao [1,5]

[1] *State Key Laboratory of Phytochemistry and Plant Resources in West China,* Kunming Institute of Botany, Chinese Academy of Sciences, Kunming 650201, *China; caimin@mail.kib.ac.cn (M.C.); zanghaojing@mail.kib.ac.cn (H.-J.Z.); tangxh1063@gmail.com (X.-H.T.); crystal_yanying@126.com (Y.Y.); wanjiajia@mail.kib.ac.cn (J.-J.W.); pmy3419@126.com (M.-Y.P.); lianghong@mail.kib.ac.cn (H.L.); liulin@mail.kib.ac.cn (L.L.); guofeng@mail.kib.ac.cn (F.G.); haoxj@mail.kib.ac.cn (X.-J.H.)*

[2] School of Life Sciences, Yunnan University, Kunming 650091, *China; pjzhao@ynu.edu.cn*

[3] *University of Chinese Academy of Sciences, Beijing 100049, China; baixl@cib.ac.cn*

[4] Chengdu Institute of Biology, Chinese Academy of Sciences, Chengdu 610041, *China*

[5] State Key Laboratory of Functions and Applications of Medicinal Plants, College of Pharmacy, Guizhou Provincial Engineering Technology Research Center for Chemical Drug R&D, Guizhou Medical University, Guiyang 550014, China

* *Correspondence:* liaoxun@cib.amc.cn (X.L.); diyt@mail.kib.ac.cn (Y.-T.D.); Tel.: +86-28-82890402 (X.L.); +86-871-65223263 (Y.-T.D.)

† These authors contributed equally to this work.

Table of content

X-ray crystallographic data of Perforalactone L (7)... 4

Table S1. Crystal data and structure refinement for 7... 4

Chemical transformation. 5

Table S2. reaction condition of Chemical transformation.. 6

Figure S1. ^{1}H NMR spectrum of Perforalactone F (1) in $CDCl_3$.. 7

Figure S2. ^{13}C NMR spectrum of Perforalactone F (1) in $CDCl_3$... 7

Figure S3. ^{1}H-^{1}H COSY spectrum of Perforalactone F (1) in $CDCl_3$ 8

Figure S4. HSQC spectrum of Perforalactone F (1) in $CDCl_3$. 8

Figure S5. HMBC spectrum of Perforalactone F (1) in $CDCl_3$. 9

Figure S6. ROESY spectrum of Perforalactone F (1) in $CDCl_3$.. 9

Figure S7. ^{1}H NMR spectrum of Perforalactone G (2) in $CDCl_3$. 10

Figure S8. ^{13}C NMR spectrum of Perforalactone G (2) in $CDCl_3$. 10

Figure S9. ^{1}H-^{1}H COSY spectrum of Perforalactone G (2) in $CDCl_3$................................ 11

Figure S10. HSQC spectrum of Perforalactone G (2) in $CDCl_3$.. 11

Figure S11. HMBC spectrum of Perforalactone G (2) in $CDCl_3$... 12

Figure S12. ROESY spectrum of Perforalactone G (2) in $CDCl_3$. 12

Figure S13. ^{1}H NMR spectrum of Perforalactone H (3) in $CDCl_3$. 13

Figure S14. ^{13}C NMR spectrum of Perforalactone H (3) in $CDCl_3$. 13

Figure S15. ^{1}H-^{1}H COSY spectrum of Perforalactone H (3) in $CDCl_3$............................. 14

Figure S16. HSQC spectrum of Perforalactone H (3) in $CDCl_3$.. 14

Figure S17. HMBC spectrum of Perforalactone H (3) in $CDCl_3$... 15

Figure S18. ROESY spectrum of Perforalactone H (3) in $CDCl_3$. 15

Figure S19. ^{1}H NMR spectrum of Perforalactone I (4) in $CDCl_3$....................................... 16

Figure S20. ^{13}C NMR spectrum of Perforalactone I (4) in $CDCl_3$...................................... 16

Figure S21. ^{1}H-^{1}H COSY spectrum of Perforalactone I (4) in $CDCl_3$. 17

Figure S22. HSQC spectrum of Perforalactone I (4) in $CDCl_3$. 17

Figure S23. HMBC spectrum of Perforalactone I (4) in $CDCl_3$. 18

Figure S24. ROESY spectrum of Perforalactone I (4) in $CDCl_3$... 18

Figure S25. ^{1}H NMR spectrum of Perforalactone J (5) in CD_3OD. 19

Figure S26. ^{13}C NMR spectrum of Perforalactone J (5) in CD_3OD. 19

Figure S27. ^{1}H-^{1}H COSY spectrum of Perforalactone J (5) in CD_3OD............................ 20

Figure S28. HSQC spectrum of Perforalactone J (5) in CD_3OD....................................... 20

Figure S29. HMBC spectrum of Perforalactone J (5) in CD_3OD...................................... 21

Figure S30. ROESY spectrum of Perforalactone J (5) in CD_3OD. 21

Figure S31. ^{1}H NMR spectrum of Perforalactone K (6) in $CDCl_3$. 22

Figure S32. ^{13}C NMR spectrum of Perforalactone K (6) in $CDCl_3$. 22

Figure S33. ^{1}H-^{1}H COSY spectrum of Perforalactone K (6) in $CDCl_3$. 23

Figure S34. HSQC spectrum of Perforalactone K (6) in $CDCl_3$.. 23

Figure S35. HMBC spectrum of Perforalactone K (6) in $CDCl_3$....................................... 24

Figure S36. ROESY spectrum of Perforalactone K (6) in $CDCl_3$. 24

Figure S37. ^{1}H NMR spectrum of Perforalactone L (7) in $CDCl_3$. 25

Figure S38. ^{13}C NMR spectrum of Perforalactone L (7) in $CDCl_3$. 25

Figure S39. ^{1}H-^{1}H COSY spectrum of Perforalactone L (7) in $CDCl_3$. 26

Figure S40. HSQC spectrum of Perforalactone L (7) in $CDCl_3$. 26

Figure S41. HMBC spectrum of Perforalactone L (7) in $CDCl_3$. 27

Figure S42. ROESY spectrum of Perforalactone L (7) in $CDCl_3$...................................... 27

X-ray crystallographic data of Perforalactone L (7)

Single-crystals of compound **7** was obtained from CH3OH/H2O (10:1) at room temperature.

Crystal data for compound 7: $C_{19}H_{22}O_5$, M = 330.36, a = 9.5789(4) Å, b = 12.0883(5) Å, c = 14.0568(6) Å, α = 90°, β = 90°, γ = 90°, V = 1627.67(12) Å3, T = 150.(2) K, space group P212121, Z = 4, μ(Cu Kα) = 0.797 mm-1, 13248 reflections measured, 3082 independent reflections (Rint = 0.0639). The final R1 values were 0.0298 (I > 2σ(I)). The final wR(F2) values were 0.0751 (I > 2σ(I)). The final R1 values were 0.0322 (all data). The final wR(F2) values were 0.0763 (all data). The goodness of fit on F2 was 1.036. Flack parameter = 0.04(8).

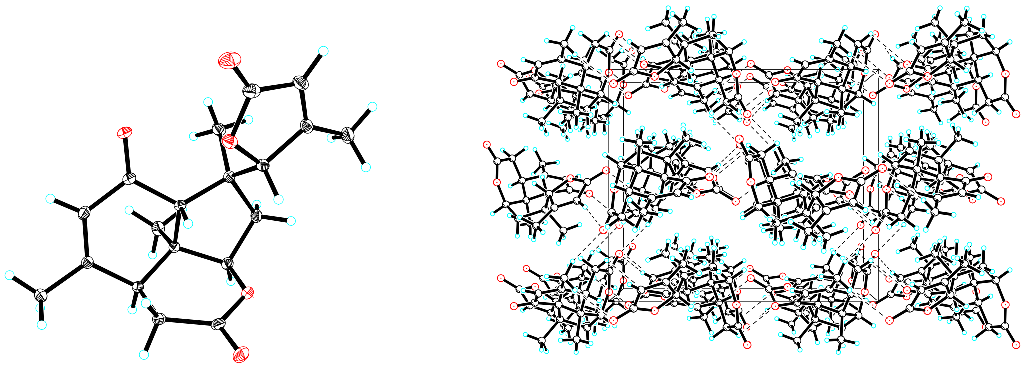

Single-crystal X-ray structure of **7**

Table S1. Crystal data and structure refinement for 7.

Identification code	global
Empirical formula	$C_{19}H_{22}O_5$
Formula weight	330.36
Temperature	150(2) K
Wavelength	1.54178 Å
Crystal system	Orthorhombic
Space group	$P2_12_12_1$

Unit cell dimensions	a = 9.5789(4) Å	a = 90°.
	b = 12.0883(5) Å	b = 90°.
	c = 14.0568(6) Å	g = 90°.
Volume	1627.67(12) Å3	
Z	4	
Density (calculated)	1.348 Mg/m^3	
Absorption coefficient	0.797 mm^{-1}	
F(000)	704	
Crystal size	0.630 x 0.400 x 0.020 mm^3	
Theta range for data collection	4.83 to 70.22°.	
Index ranges	-11<=h<=11, -14<=k<=14, -17<=l<=15	
Reflections collected	13248	
Independent reflections	3082 [R(int) = 0.0639]	
Completeness to theta = 70.22°	99.7 %	
Absorption correction	Semi-empirical from equivalents	
Max. and min. transmission	0.98 and 0.70	
Refinement method	Full-matrix least-squares on F^2	
Data / restraints / parameters	3082 / 0 / 221	
Goodness-of-fit on F^2	1.036	
Final R indices [I>2sigma(I)]	R1 = 0.0298, wR2 = 0.0751	
R indices (all data)	R1 = 0.0322, wR2 = 0.0763	
Absolute structure parameter	0.04(8)	
Largest diff. peak and hole	0.209 and -0.158 e.Å^{-3}	

Chemical transformation.

Compound **8** was obtained by oxidative conversion of natural product Perforalactone B via Bayer-Weilig.

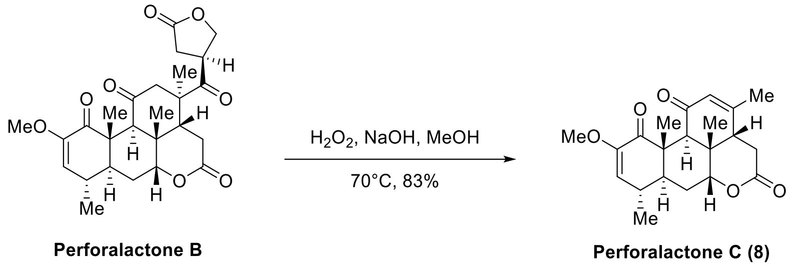

Yield 83%. To a solution of Perforalactone B (1) (20 mg, 42.32 μmol) in Chromatographic methanol was (20 ml) added H_2O_2(30%, 10 mml, 95.65 μmol) at that temperature, After being warmed to 60°C, NaOH(aq) (2.50 mol/L, 136 μml, 36.40 μmol) was added. After stirring for 30 min at 70 °C, the reaction mixture was quenched with sat. $Na_2S_2O_4$, and extracted with EtOAc. The organic phase was washed with brine, dried over Na_2SO_4 filtered, and evaporated to dryness *in vacuo*. The crude product was purified by silica gel column chromatography to afford Compound **8**.

Of course, a number of reaction conditions were screened in order to successfully realize this chemical transformation.

Table S2. reaction condition of Chemical transformation.

Entry	reagent/equiv	temp	yield
1	K_2CO_3/1 equiv	r.t.	no rxn
2	K_2CO_3/over equiv	r.t.	no rxn
3	K_2CO_3/1 equiv	50℃	no rxn
4	K_2CO_3, O_2/1 equiv	r.t.	no rxn
5	NaOH/1 equiv	r.t.	no rxn
6	K_2CO_3/1 equiv+3-Chlorobenzoic acid/1equiv	r.t.	no rxn
7	NaOH/0.86 equiv+H_2O_2/2.5	70 °C	no rxn

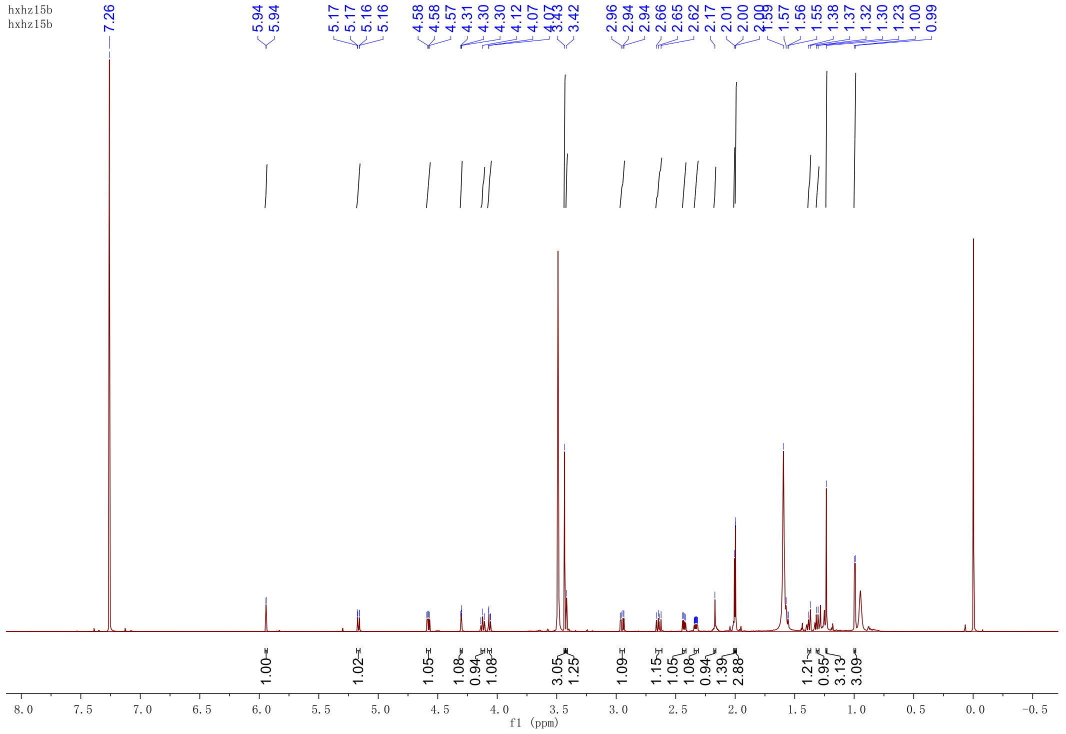

Figure S1. ^{1}H NMR spectrum of Perforalactone F (1) in $CDCl_3$.

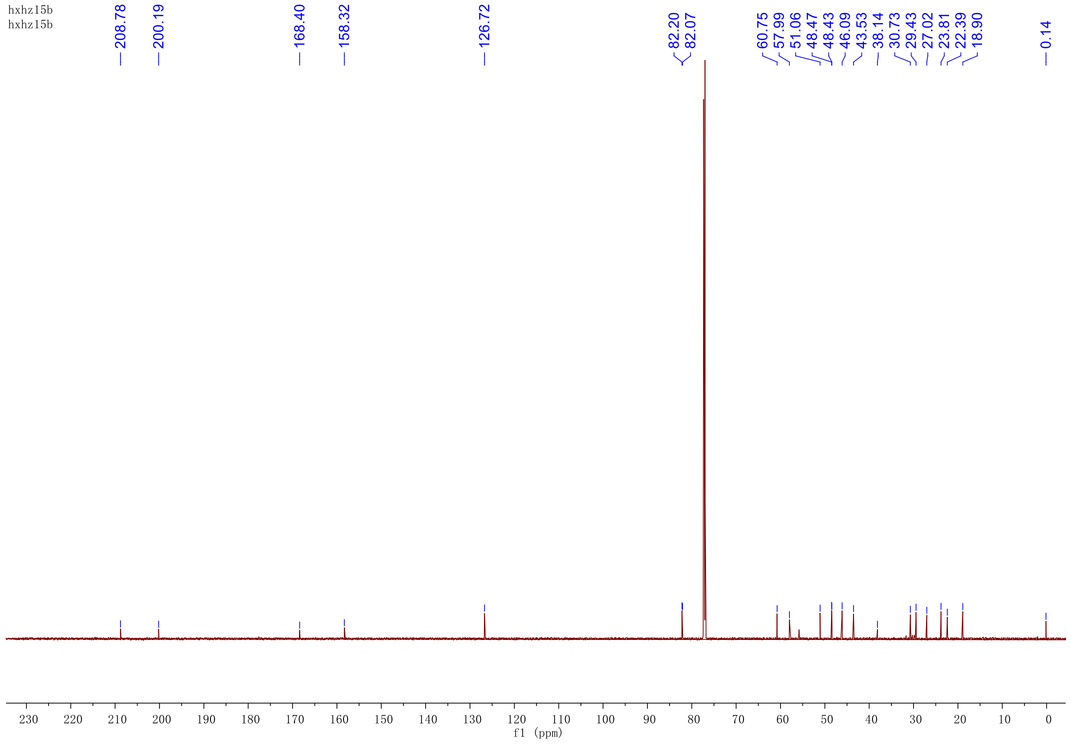

Figure S2. ^{13}C NMR spectrum of Perforalactone F (1) in $CDCl_3$.

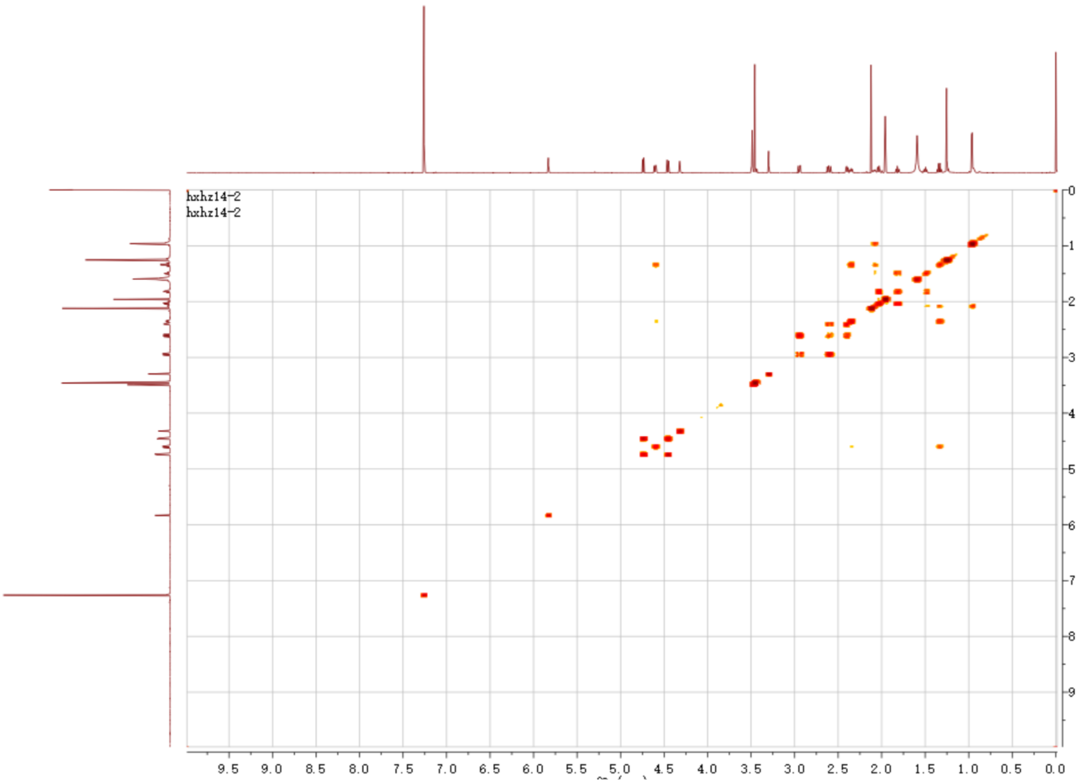

Figure S3. ^1H-^1H COSY spectrum of Perforalactone F (1) in $CDCl_3$

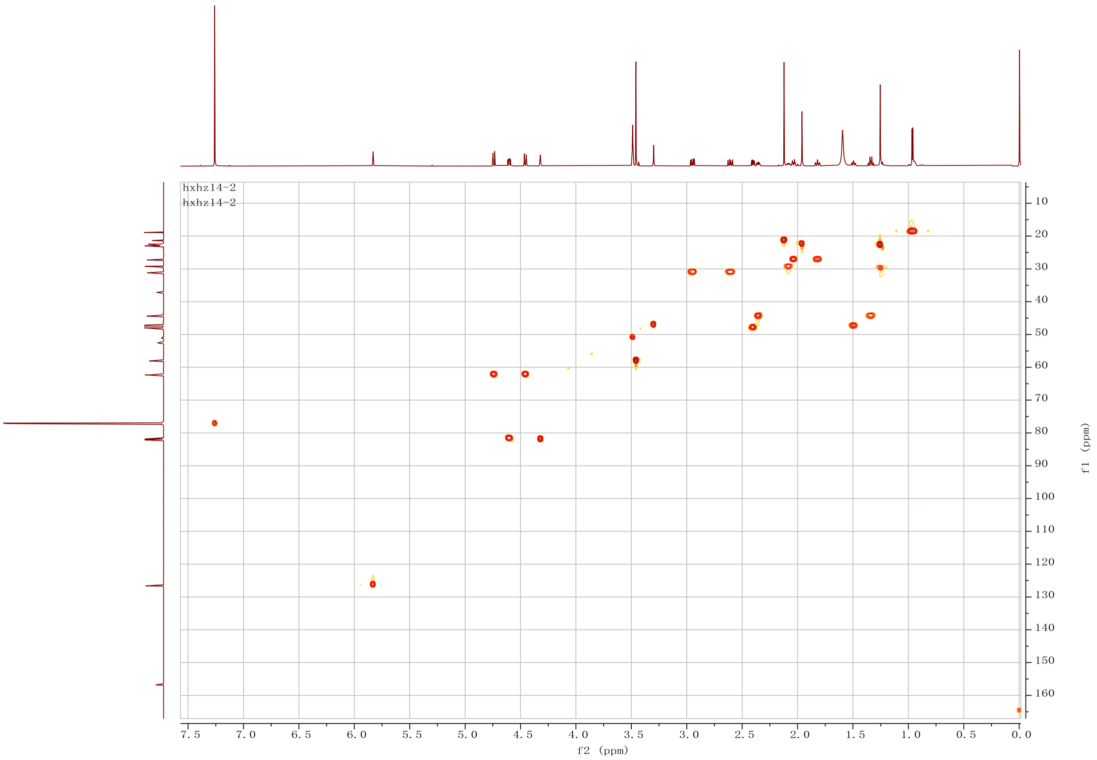

Figure S4. HSQC spectrum of Perforalactone F (1) in $CDCl_3$.

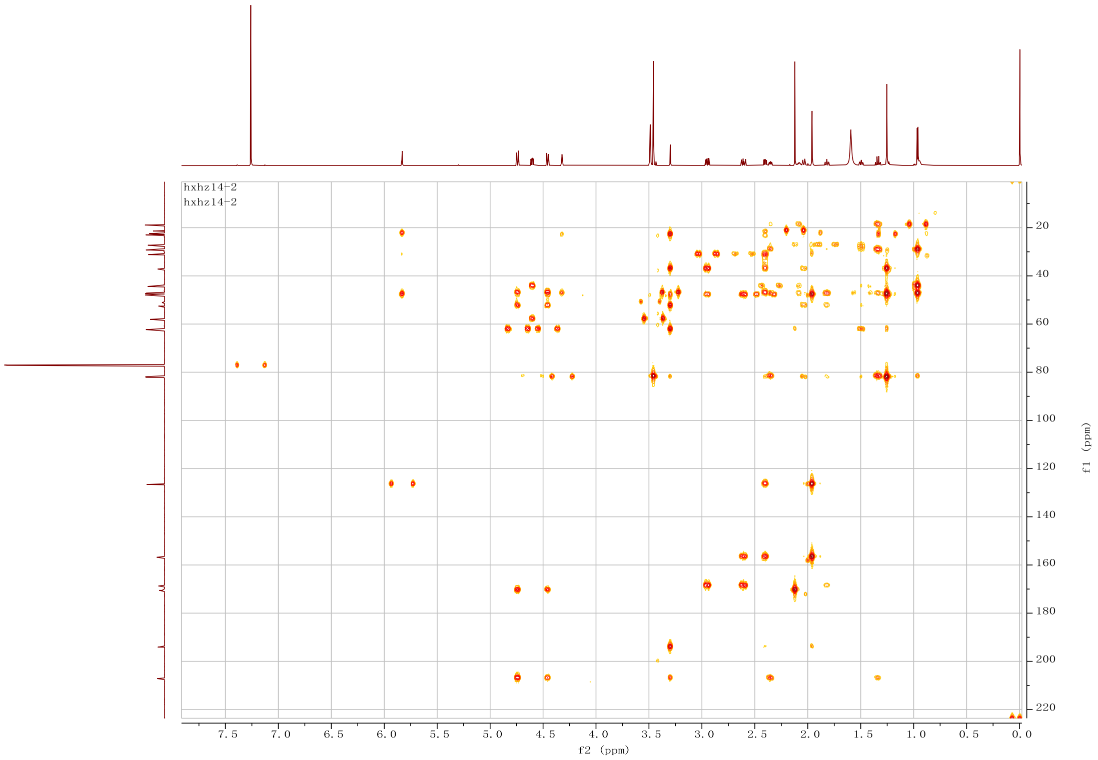

Figure S5. HMBC spectrum of Perforalactone F (1) in $CDCl_3$.

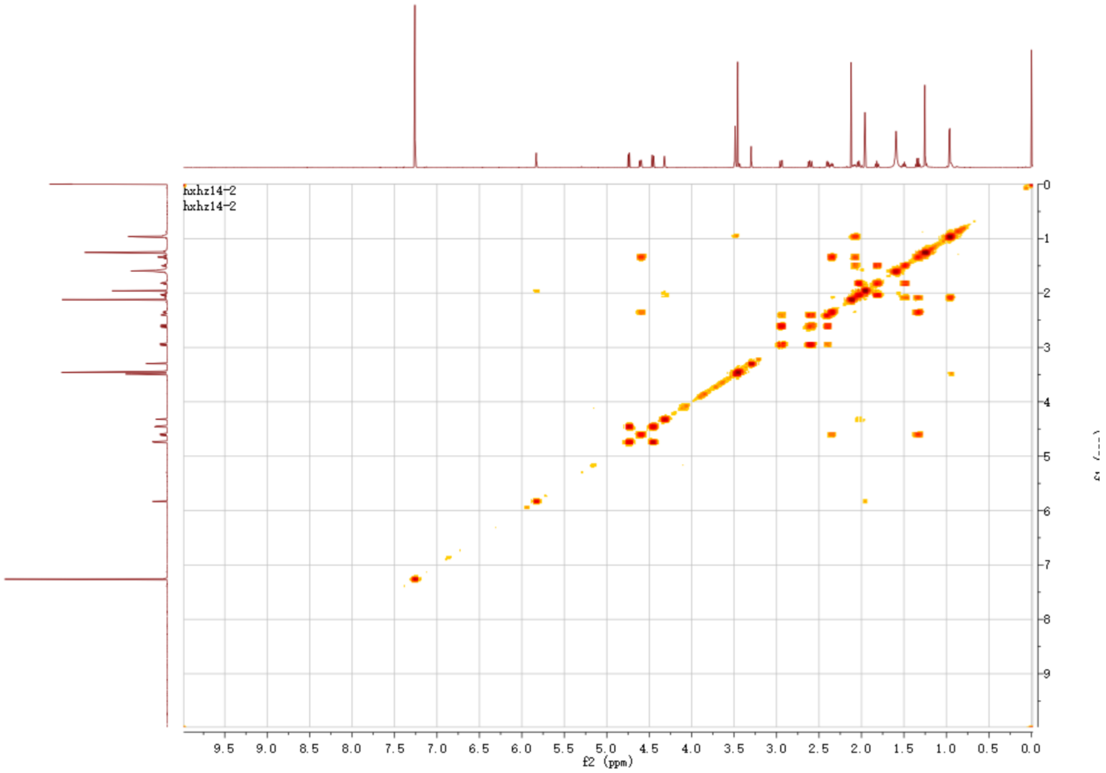

Figure S6. ROESY spectrum of Perforalactone F (1) in $CDCl_3$.

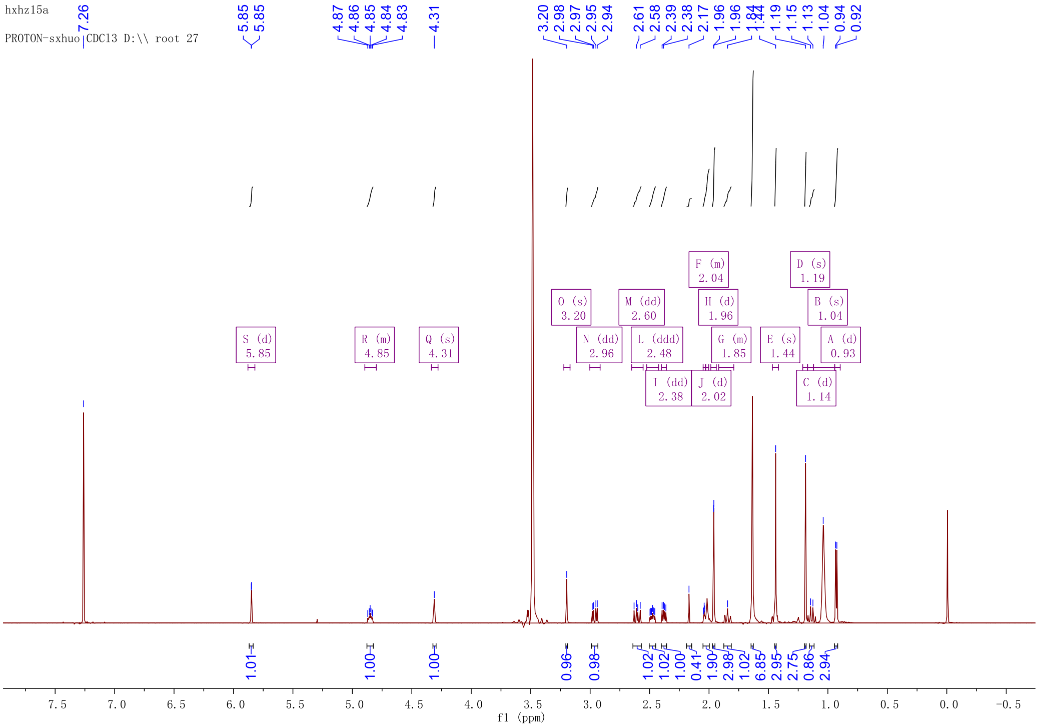

Figure S7. ^{1}H NMR spectrum of Perforalactone G (2) in CDCl₃.

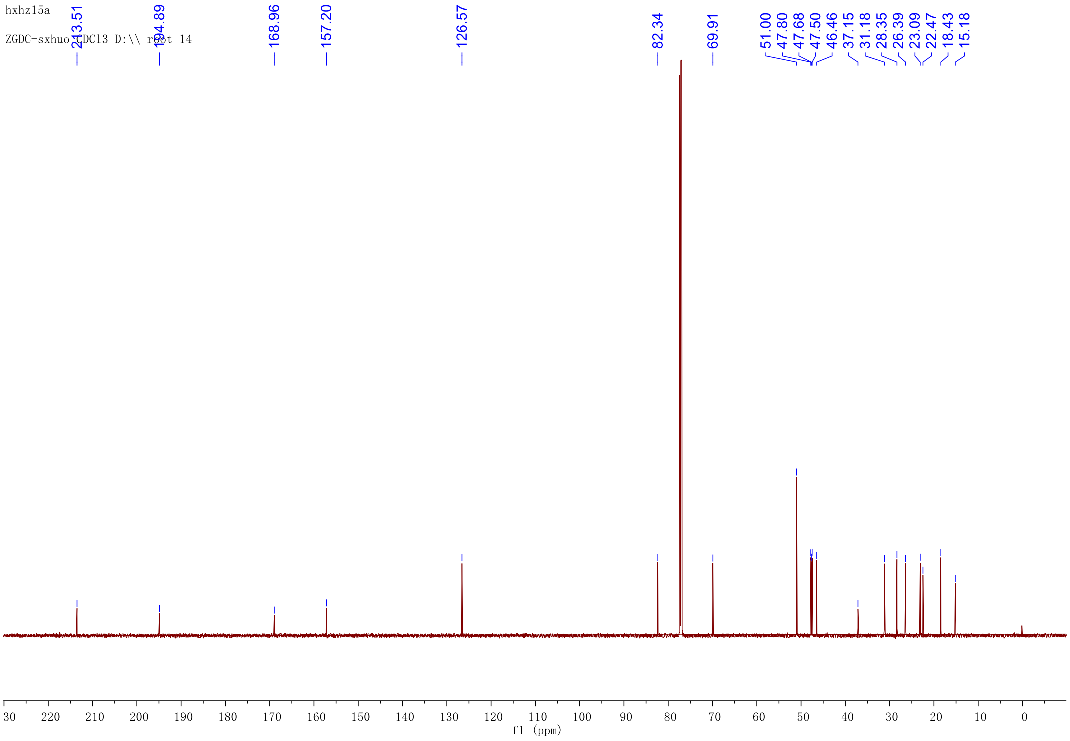

Figure S8. ^{13}C NMR spectrum of Perforalactone G (2) in CDCl₃.

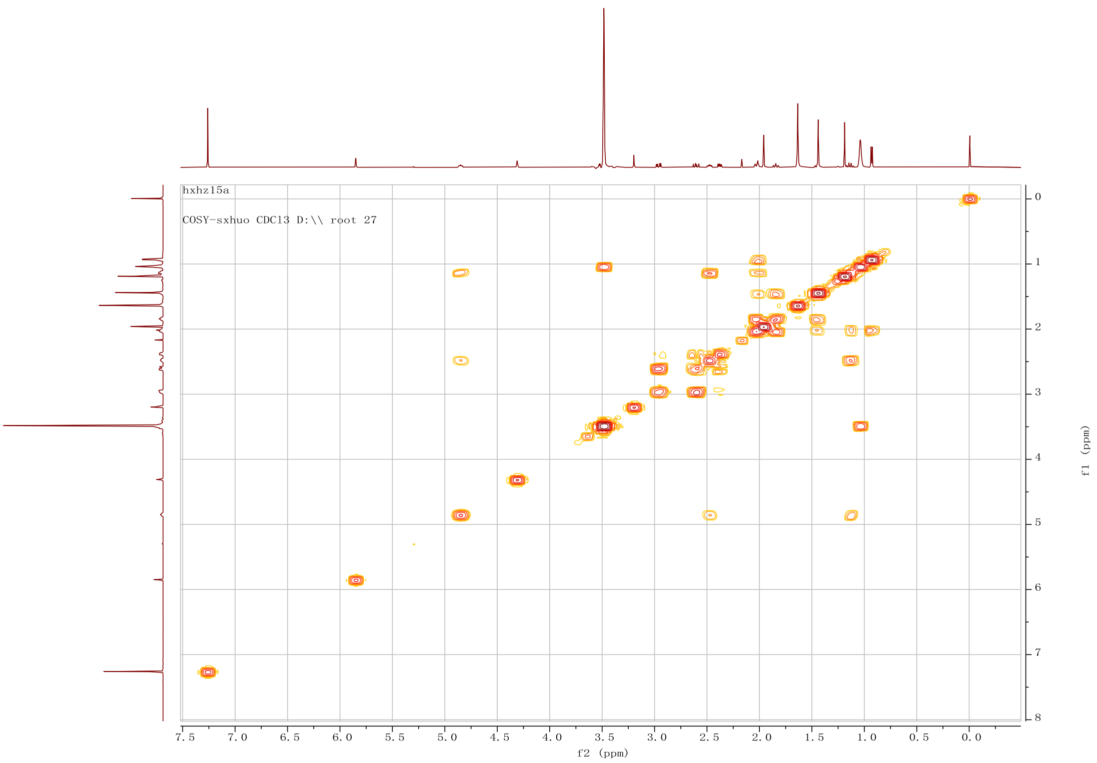

Figure S9. ^{1}H-^{1}H COSY spectrum of Perforalactone G (2) in $CDCl_3$.

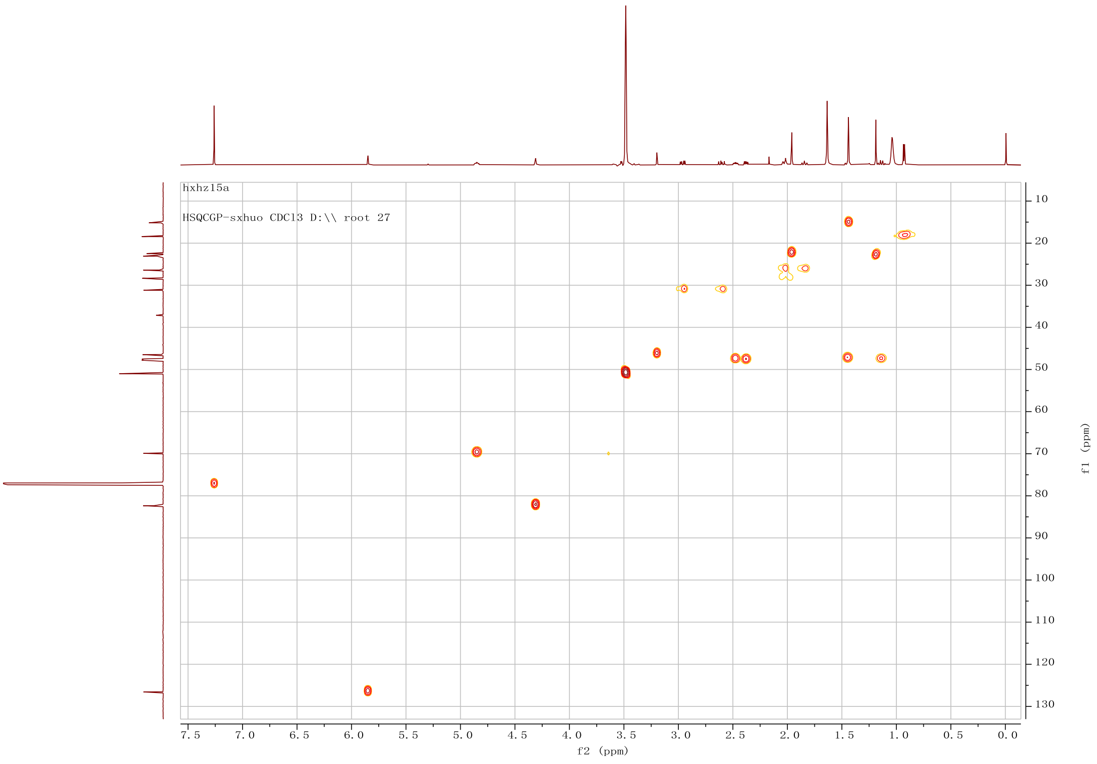

Figure S10. HSQC spectrum of Perforalactone G (2) in $CDCl_3$.

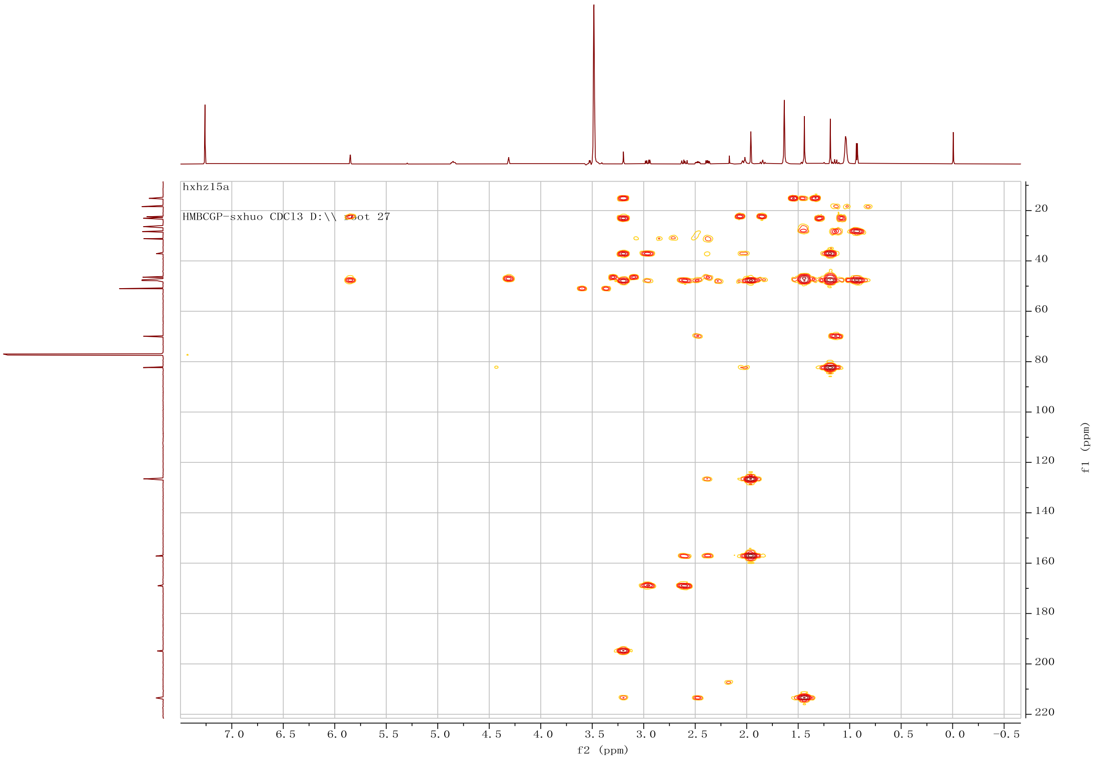

Figure S11. HMBC spectrum of Perforalactone G (2) in $CDCl_3$.

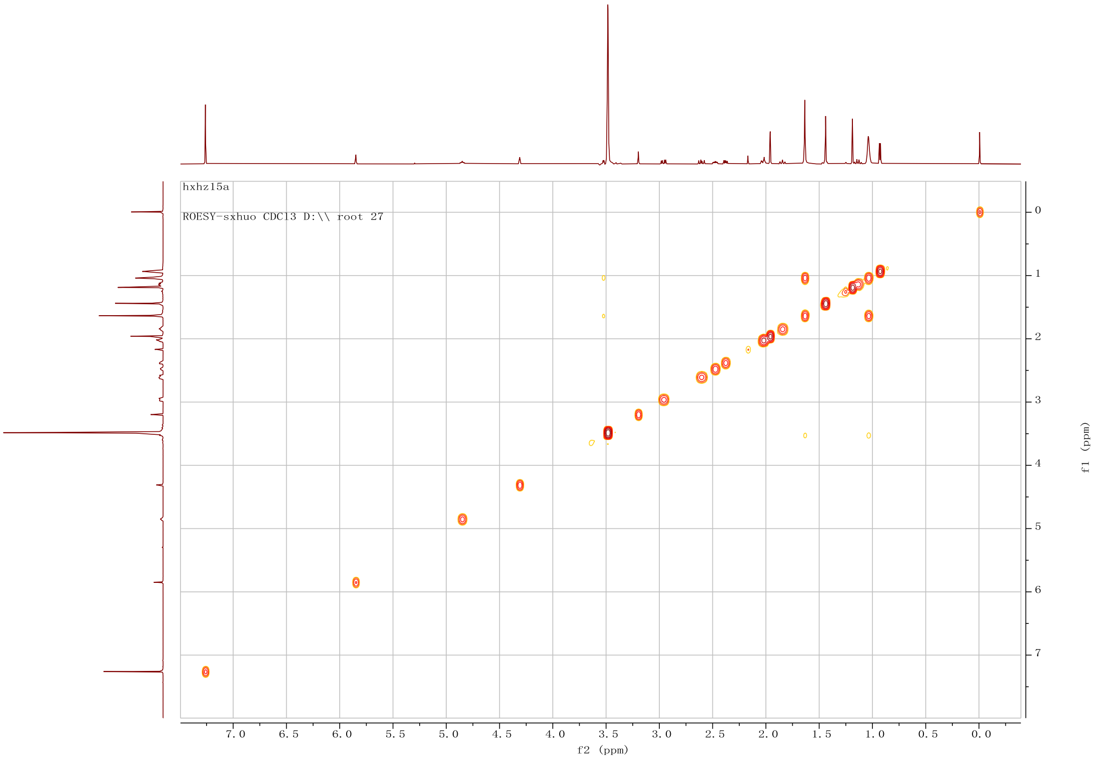

Figure S12. ROESY spectrum of Perforalactone G (2) in $CDCl_3$.

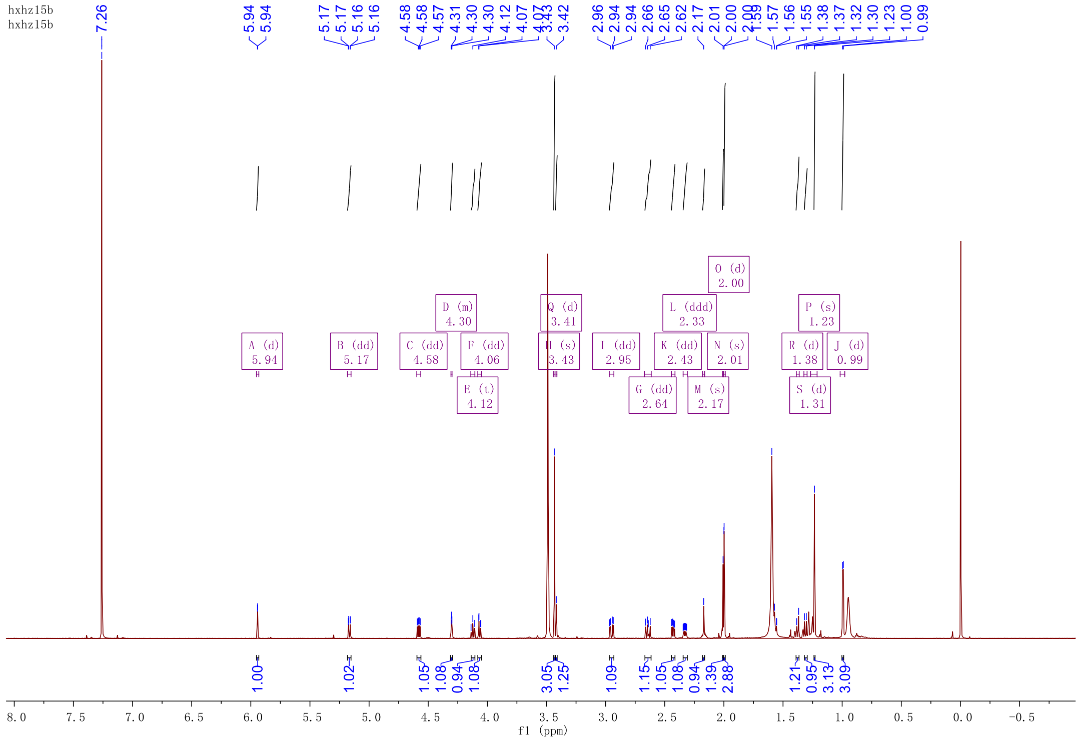

Figure S13. **^{1}H NMR spectrum of Perforalactone H (3) in $CDCl_3$.**

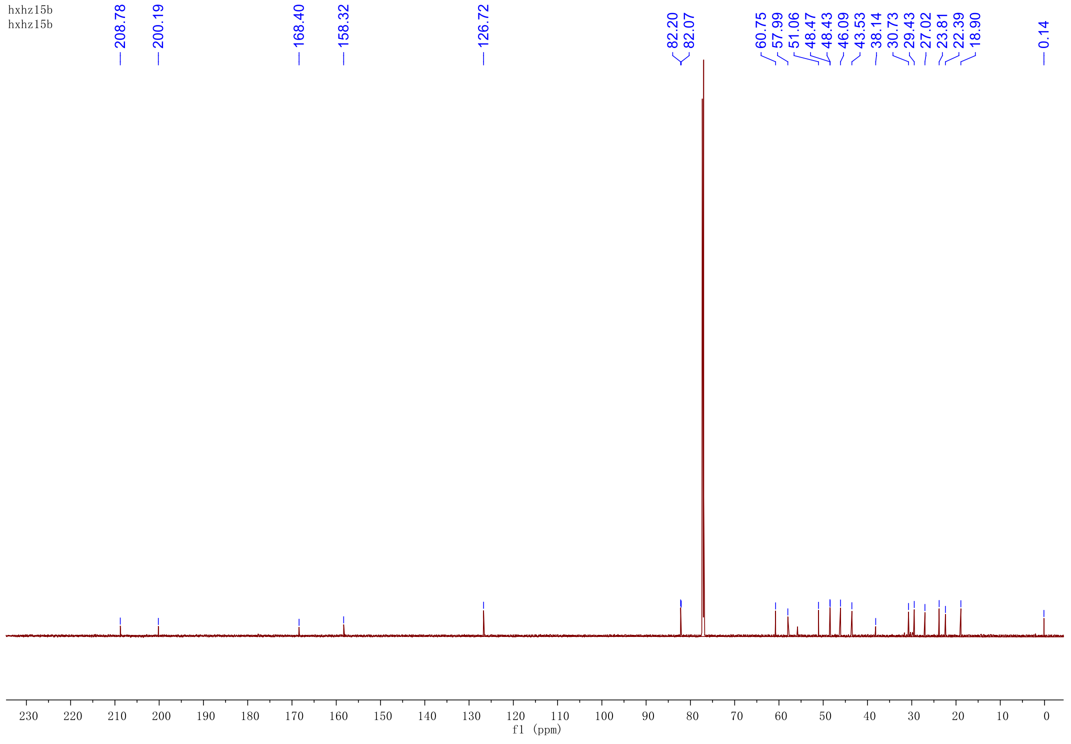

Figure S14. **^{13}C NMR spectrum of Perforalactone H (3) in $CDCl_3$.**

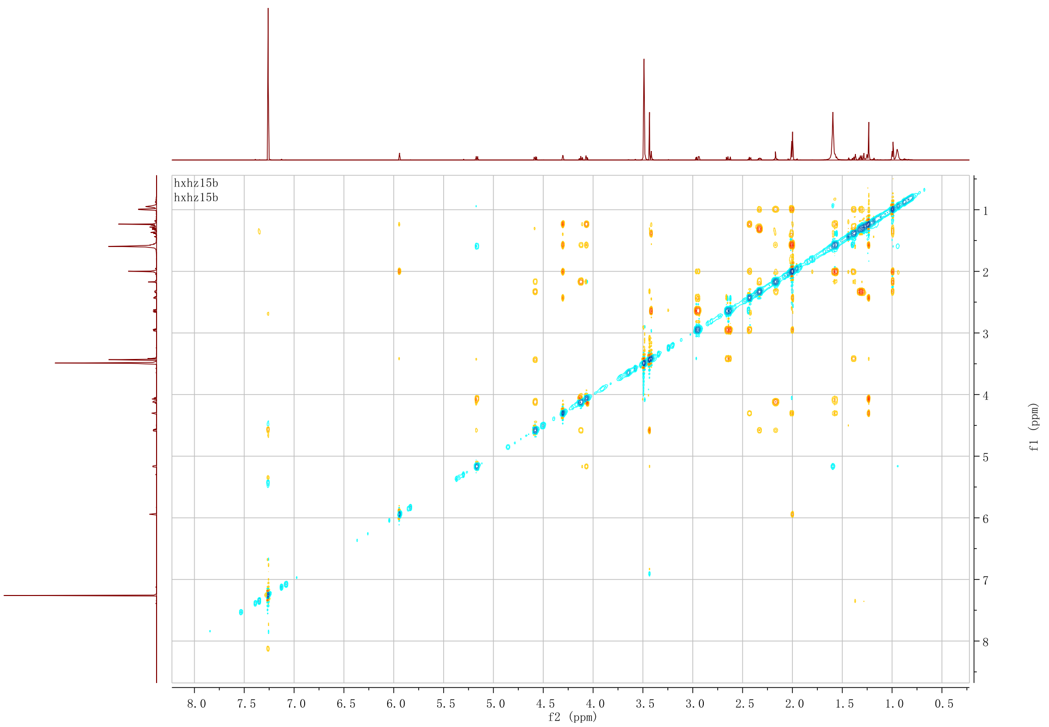

Figure S15. ^{1}H-^{1}H COSY spectrum of Perforalactone H (3) in $CDCl_3$.

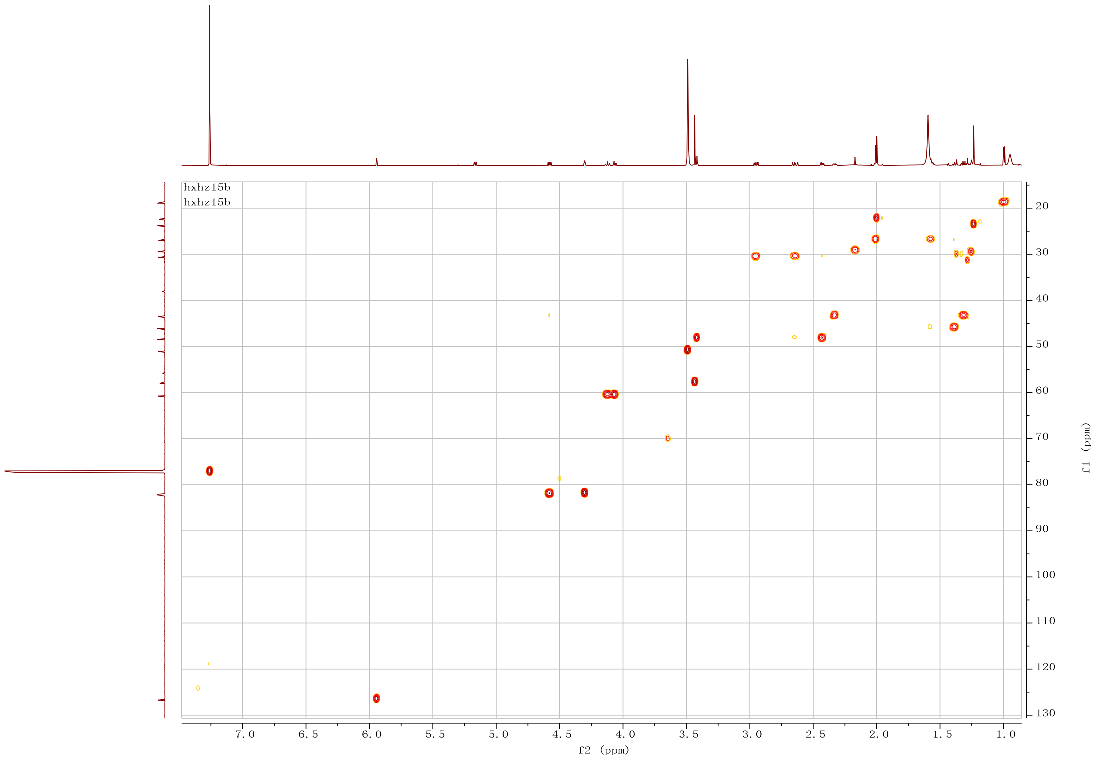

Figure S16. HSQC spectrum of Perforalactone H (3) in $CDCl_3$.

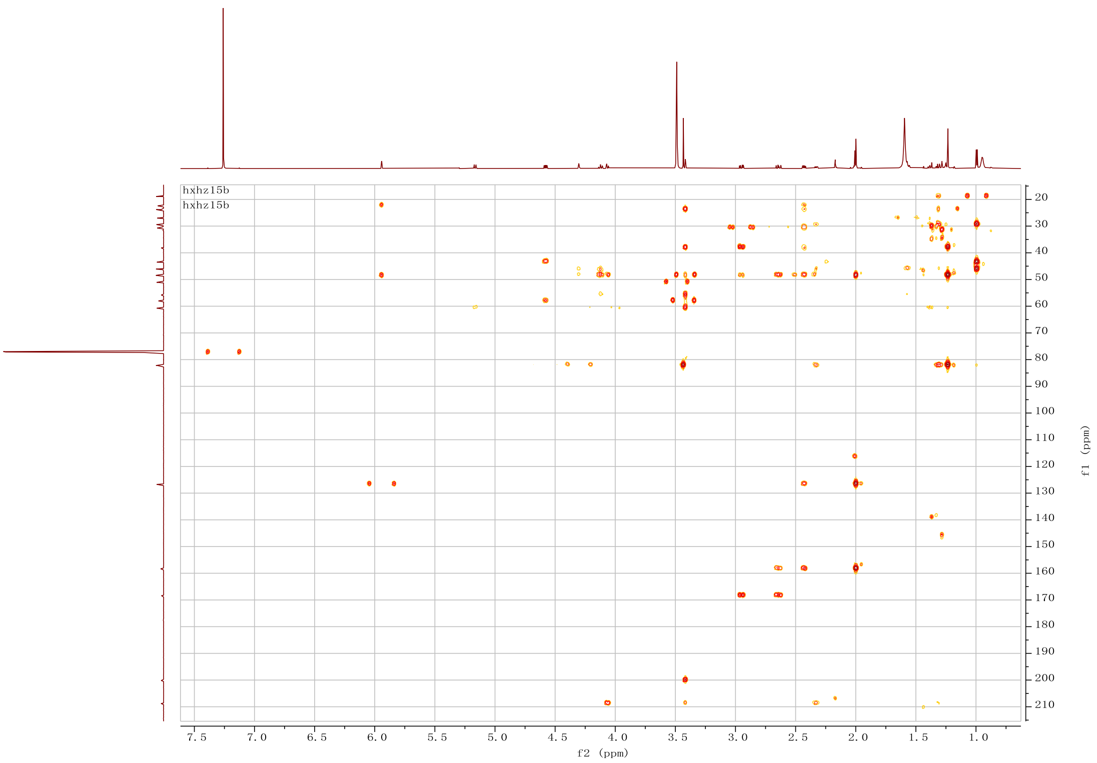

Figure S17. HMBC spectrum of Perforalactone H (3) in $CDCl_3$.

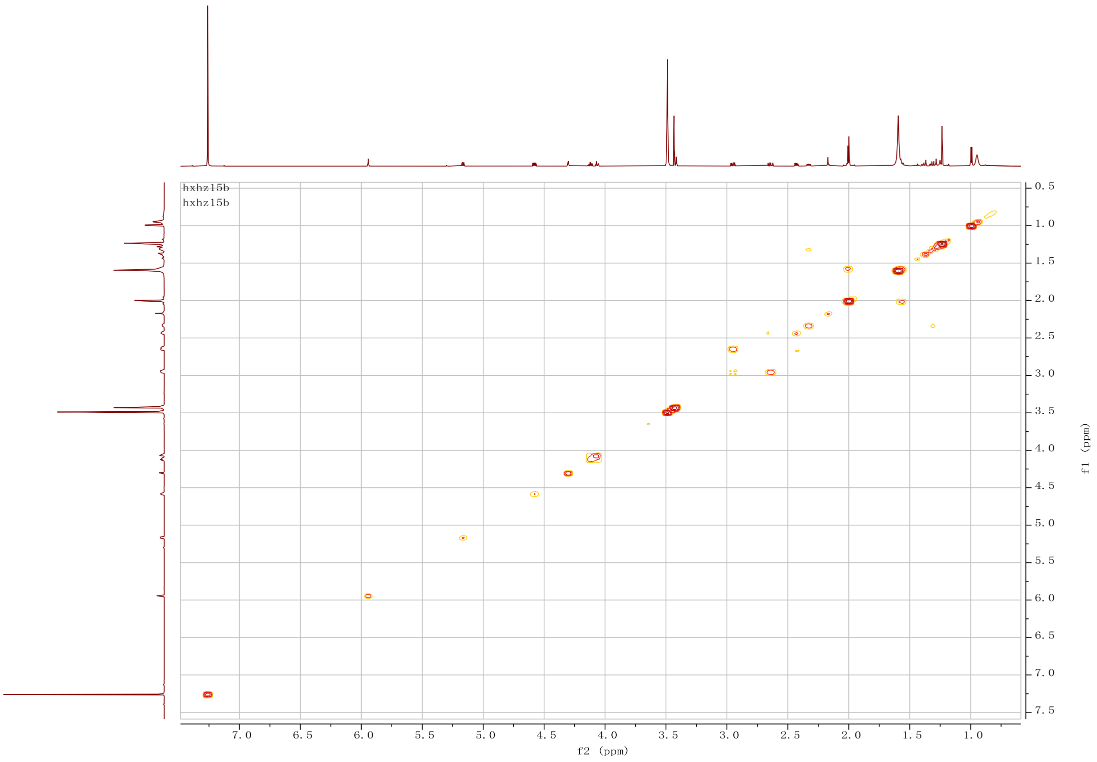

Figure S18. ROESY spectrum of Perforalactone H (3) in $CDCl_3$.

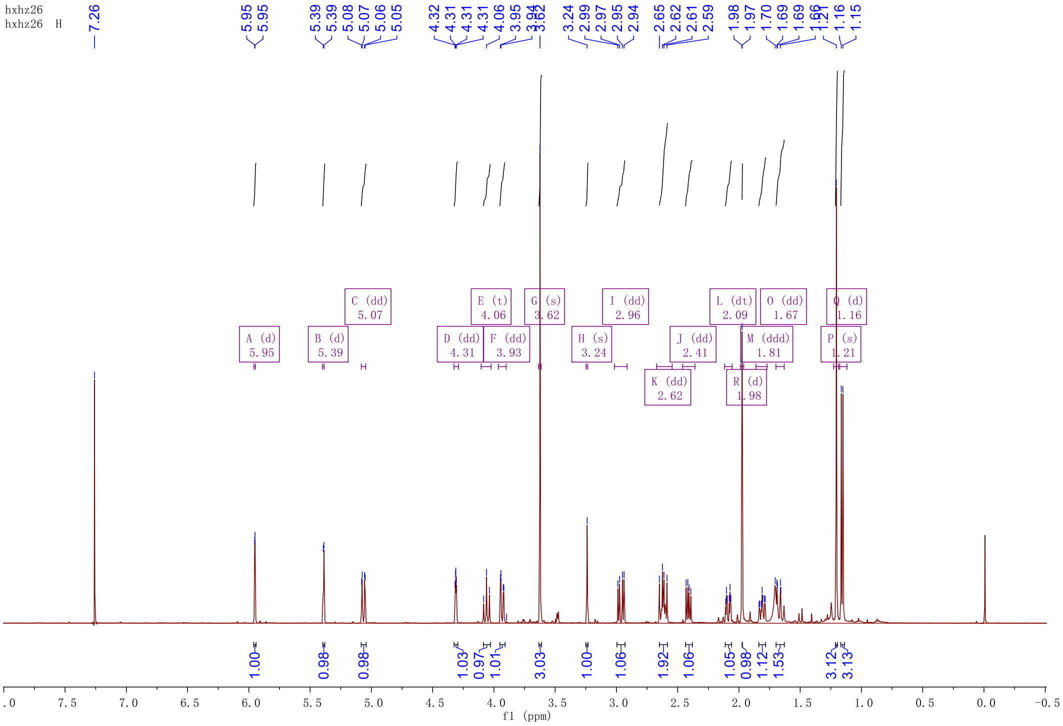

Figure S19. ^{1}H NMR spectrum of Perforalactone I (4) in $CDCl_3$.

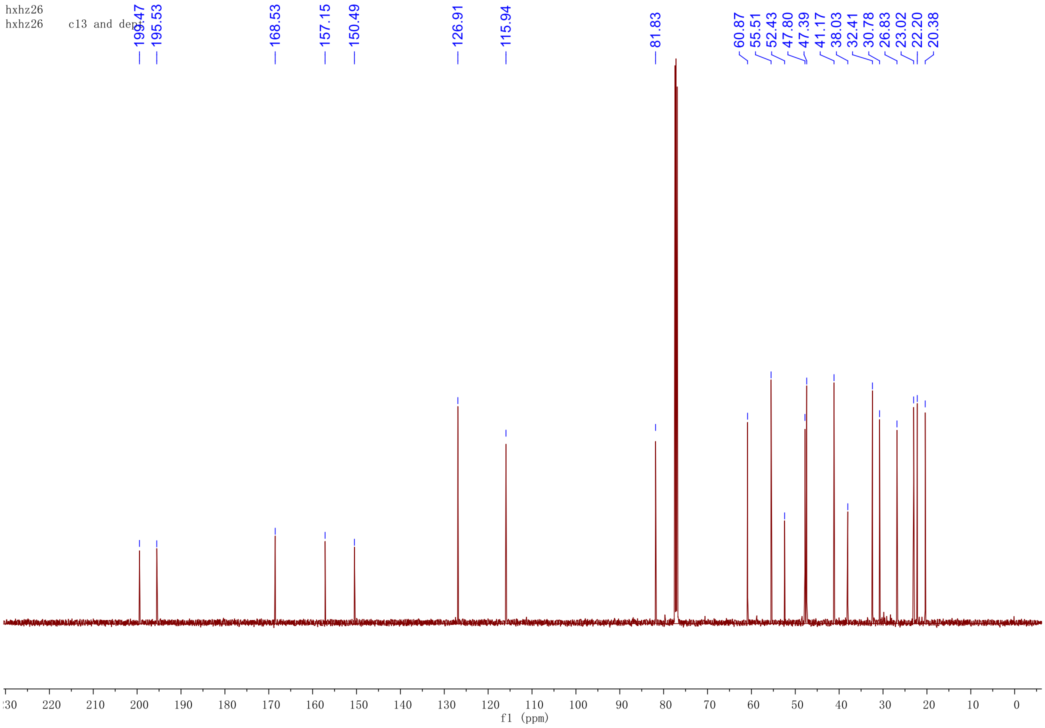

Figure S20. ^{13}C NMR spectrum of Perforalactone I (4) in $CDCl_3$.

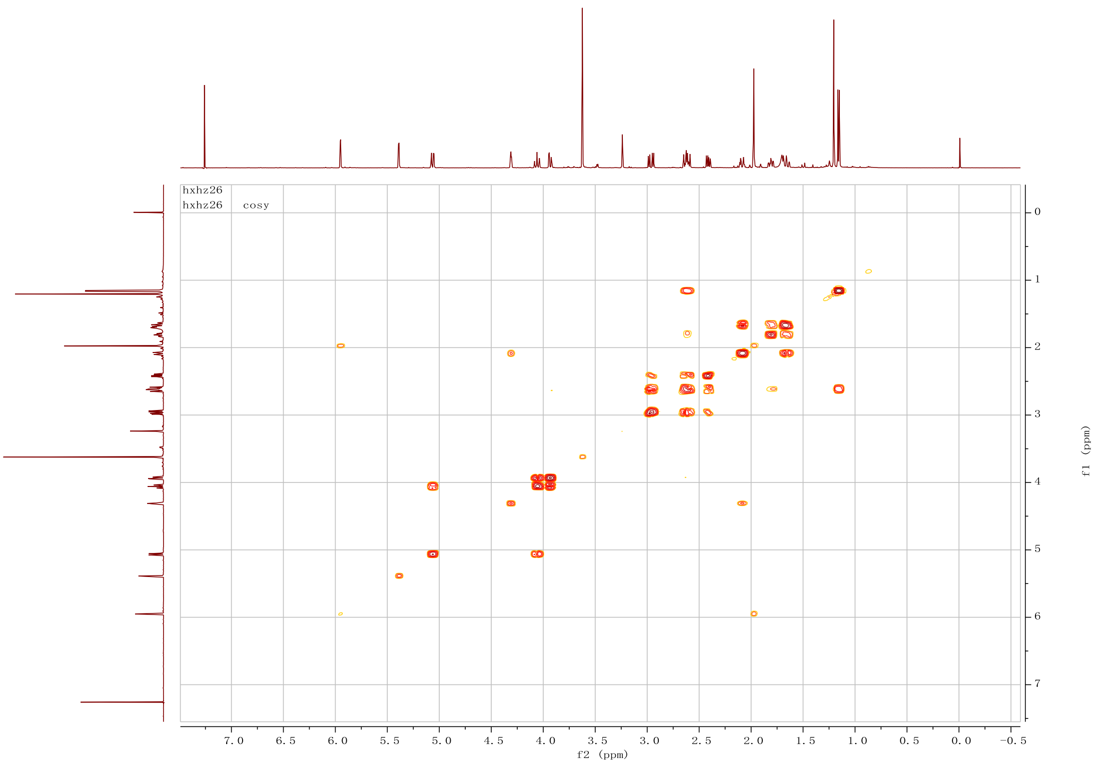

Figure S21. ^{1}H-^{1}H COSY spectrum of Perforalactone I (4) in $CDCl_3$.

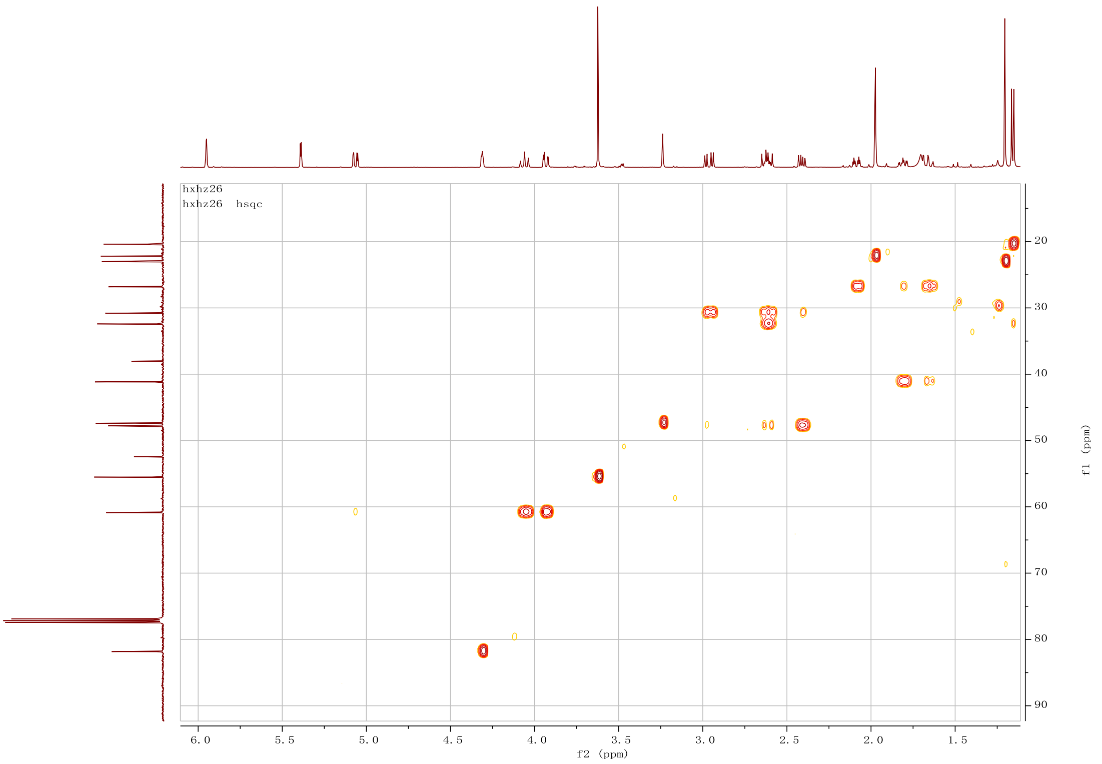

Figure S22. HSQC spectrum of Perforalactone I (4) in $CDCl_3$.

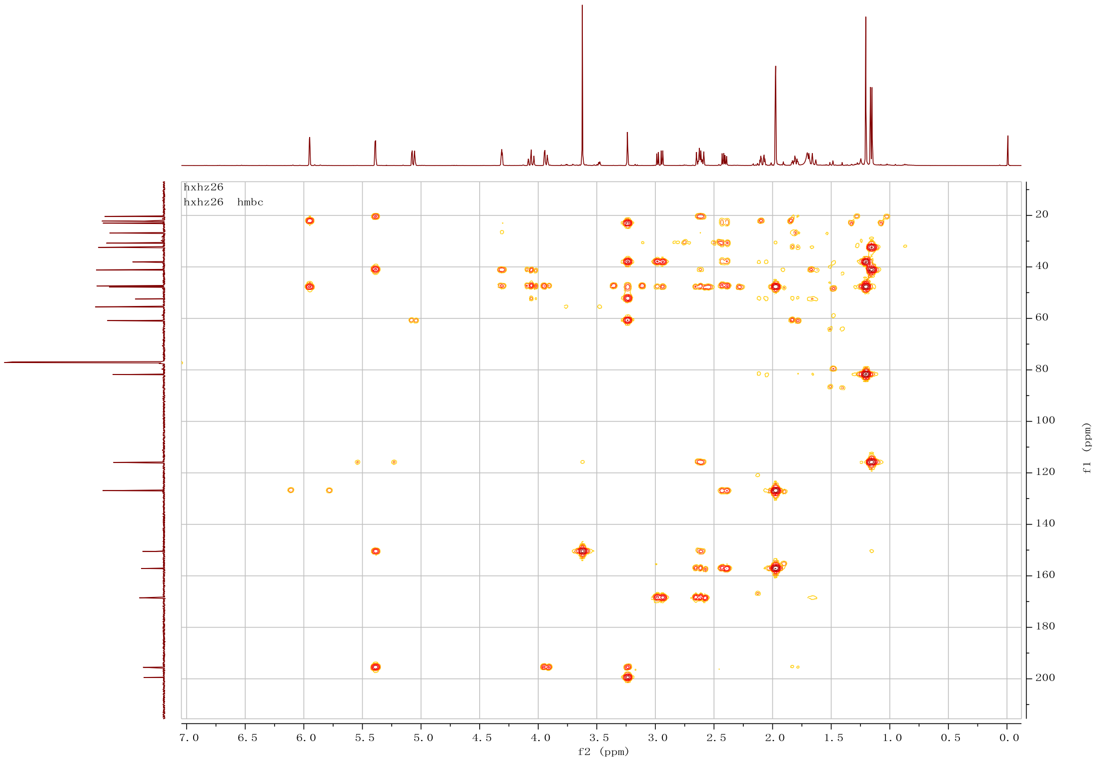

Figure S23. HMBC spectrum of Perforalactone I (4) in $CDCl_3$.

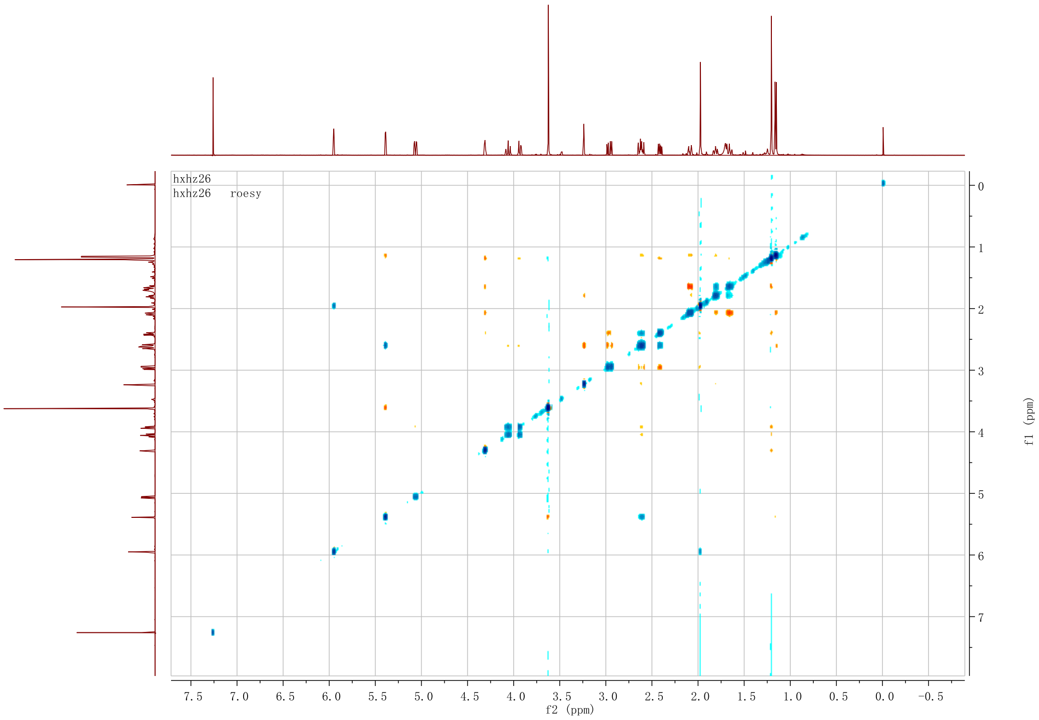

Figure S24. ROESY spectrum of Perforalactone I (4) in $CDCl_3$.

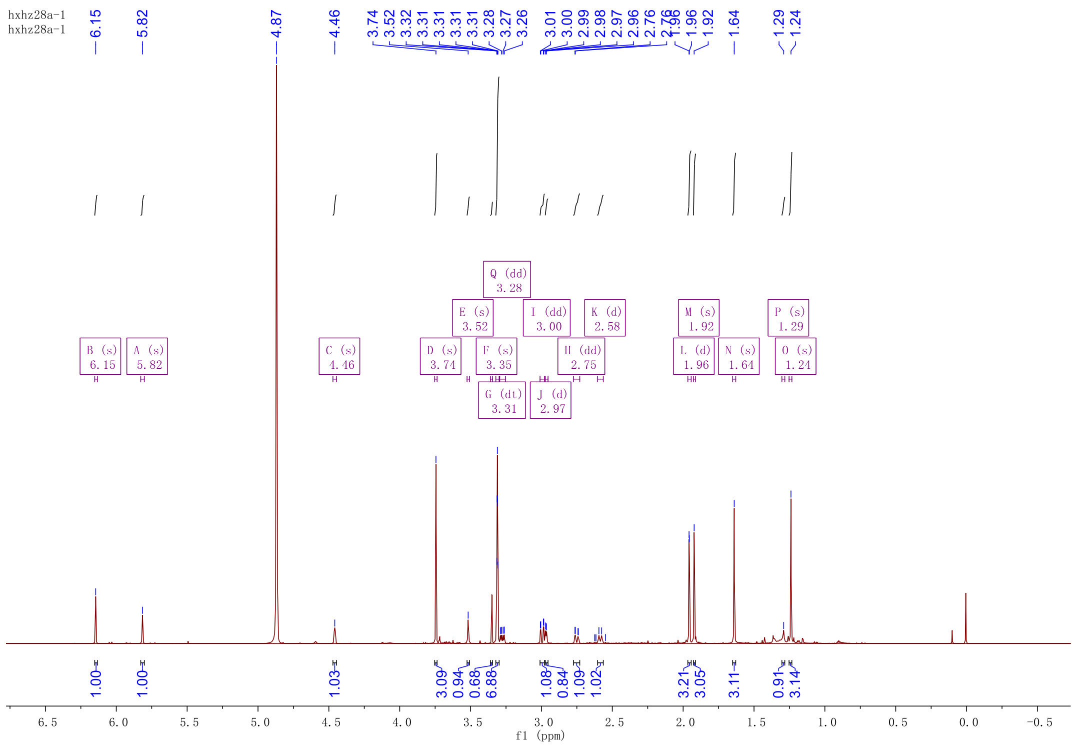

Figure S25. ^{1}H NMR spectrum of Perforalactone J (5) in CD_3OD.

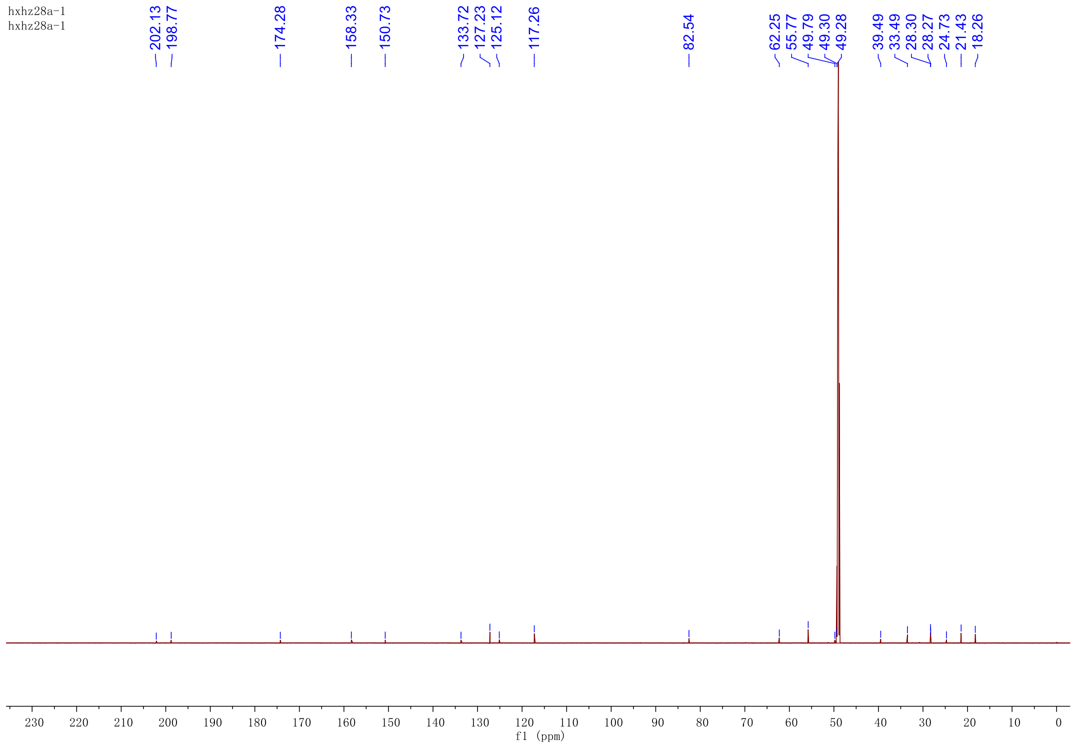

Figure S26. ^{13}C NMR spectrum of Perforalactone J (5) in CD_3OD.

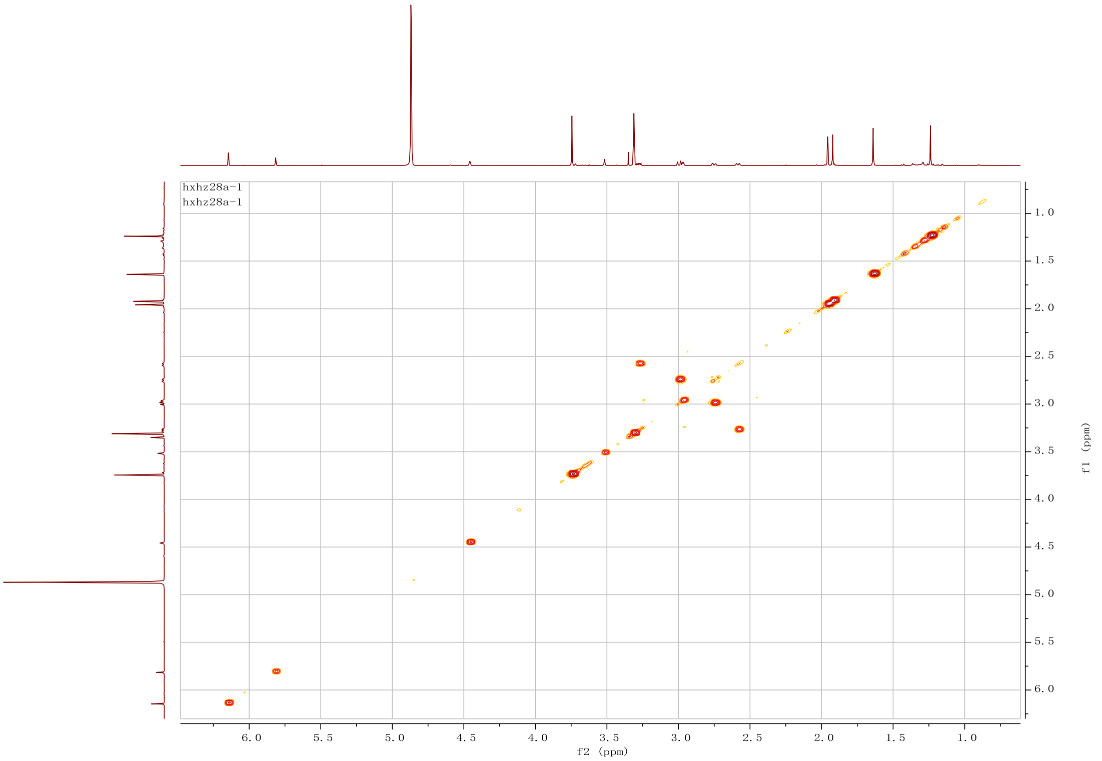

Figure S27. ^{1}H-^{1}H COSY spectrum of Perforalactone J (5) in CD_3OD.

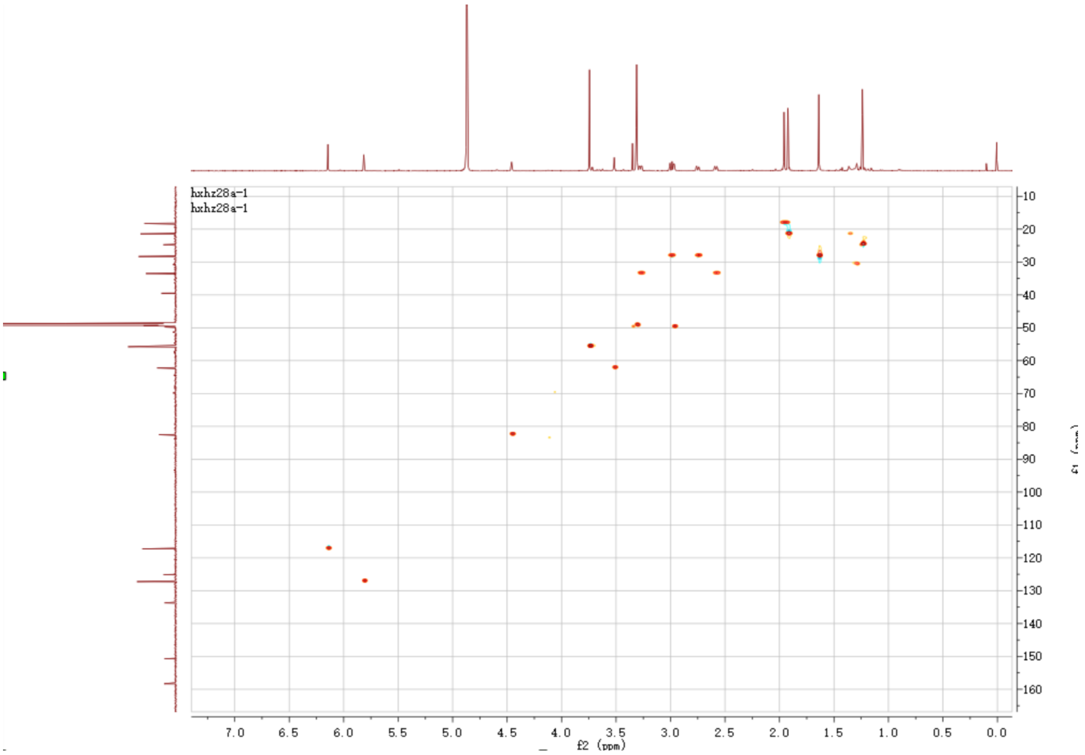

Figure S28. HSQC spectrum of Perforalactone J (5) in CD_3OD.

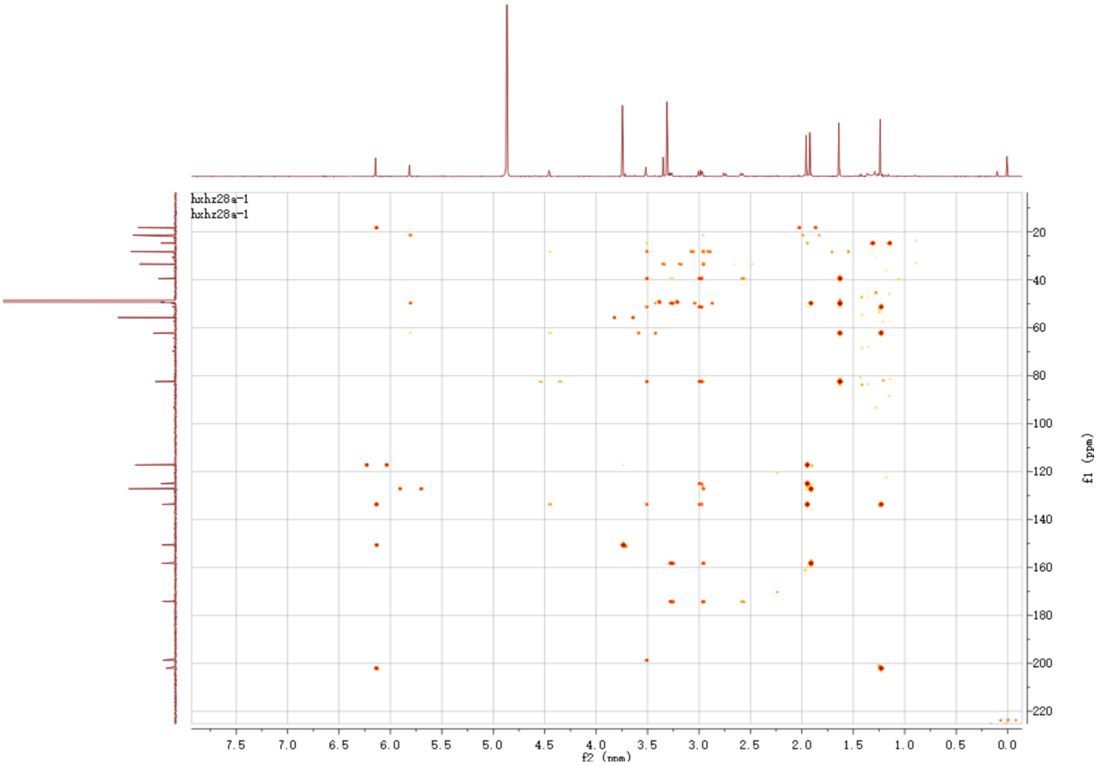

Figure S29. HMBC spectrum of Perforalactone J (5) in CD_3OD.

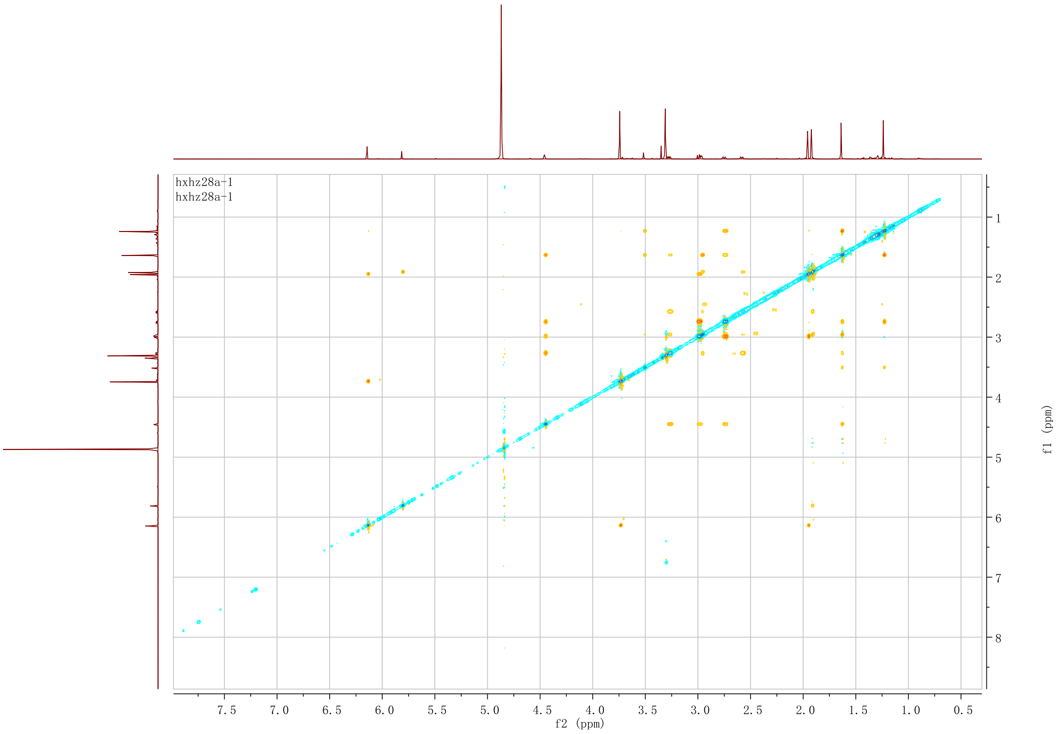

Figure S30. ROESY spectrum of Perforalactone J (5) in CD_3OD.

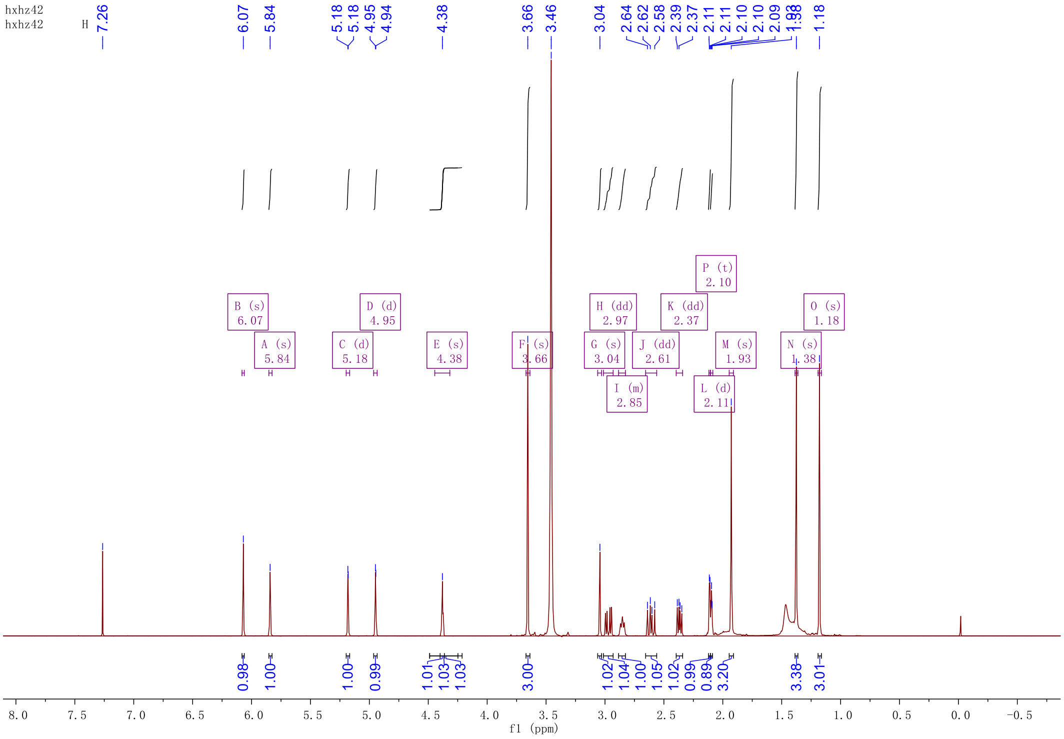

Figure S31. ^{1}H NMR spectrum of Perforalactone K (6) in $CDCl_3$.

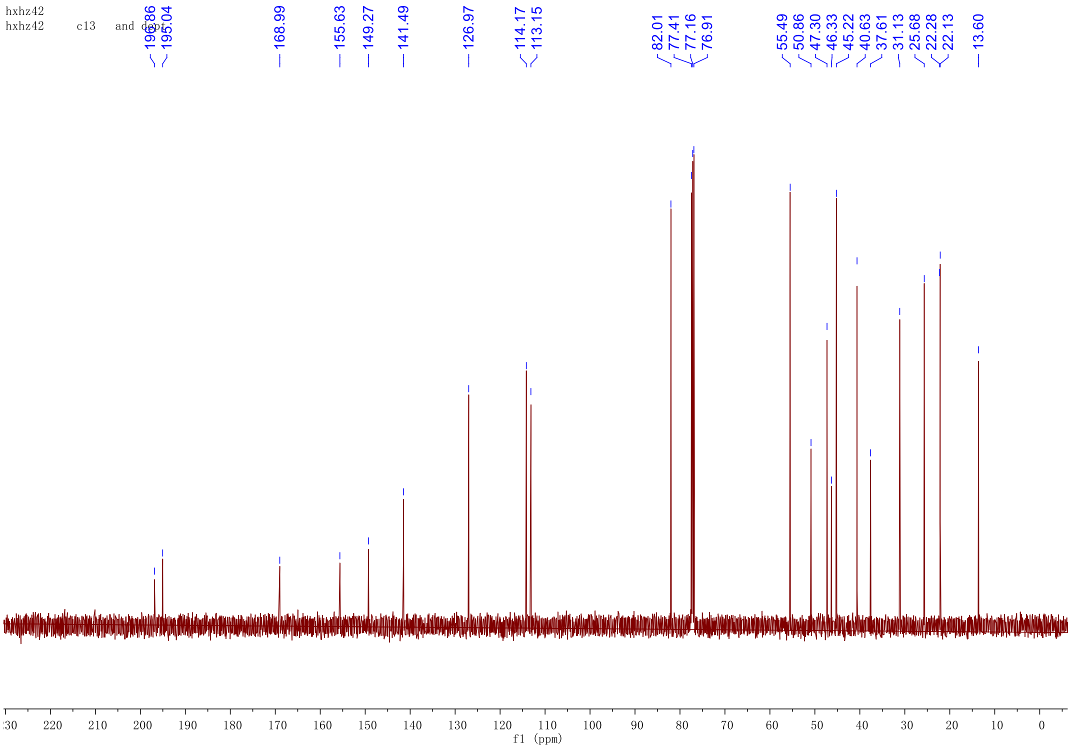

Figure S32. ^{13}C NMR spectrum of Perforalactone K (6) in $CDCl_3$.

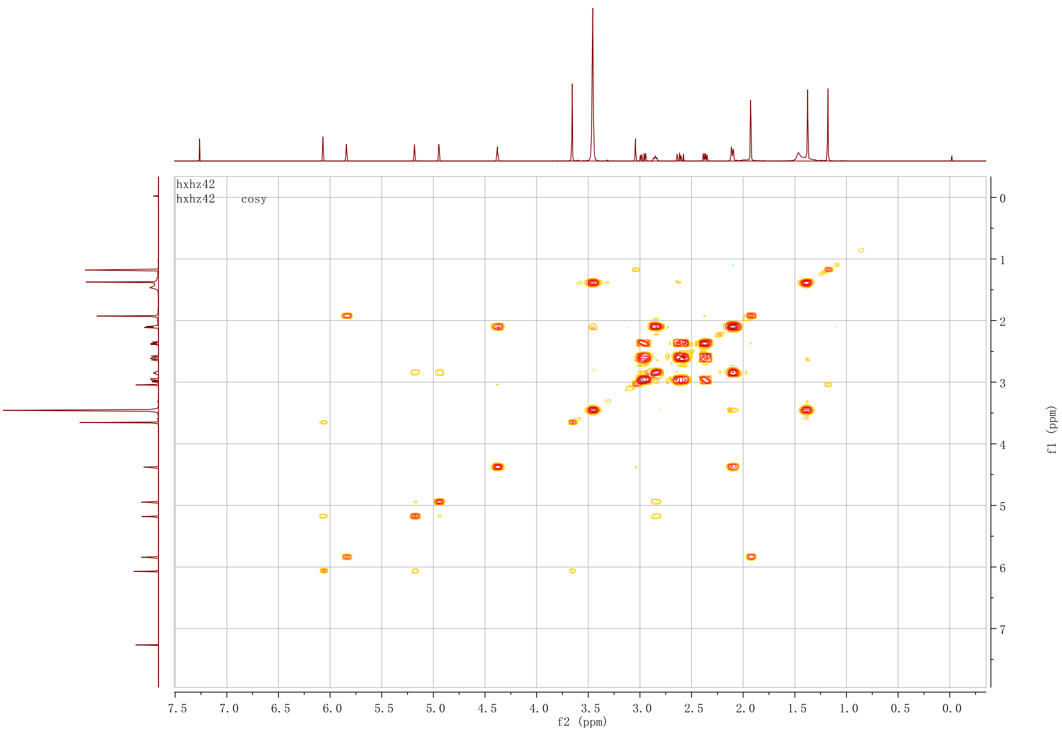

Figure S33. ^{1}H-^{1}H COSY spectrum of Perforalactone K (6) in $CDCl_3$.

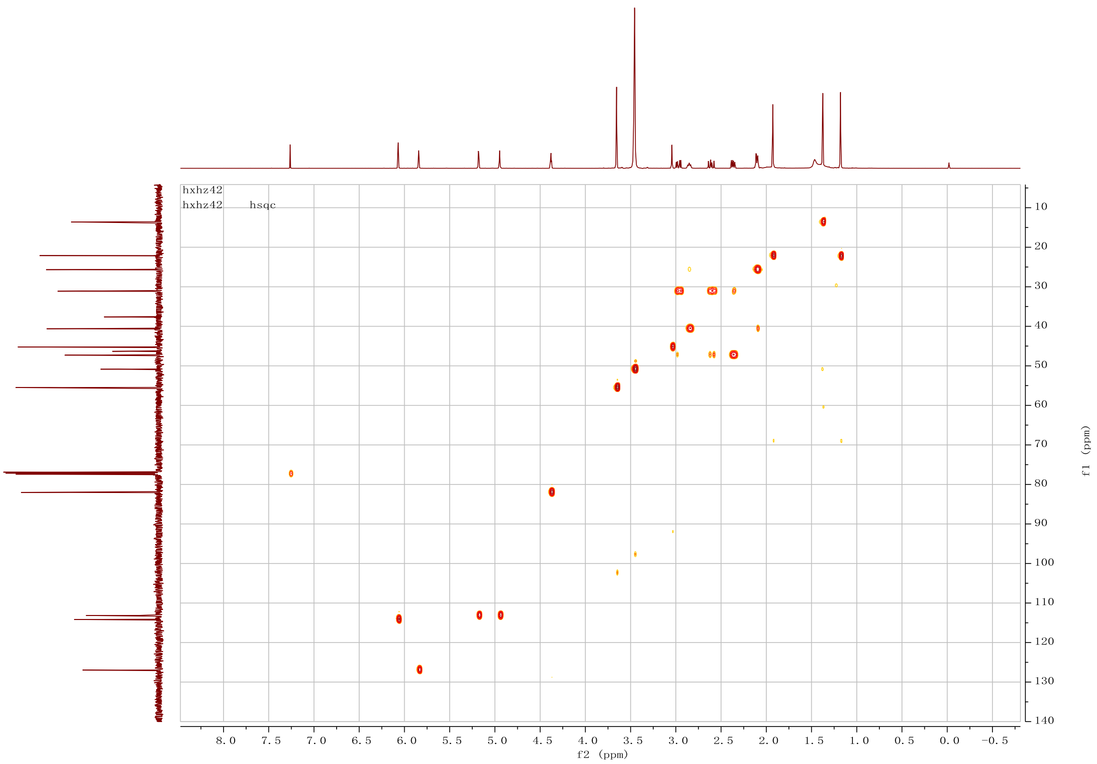

Figure S34. HSQC spectrum of Perforalactone K (6) in $CDCl_3$.

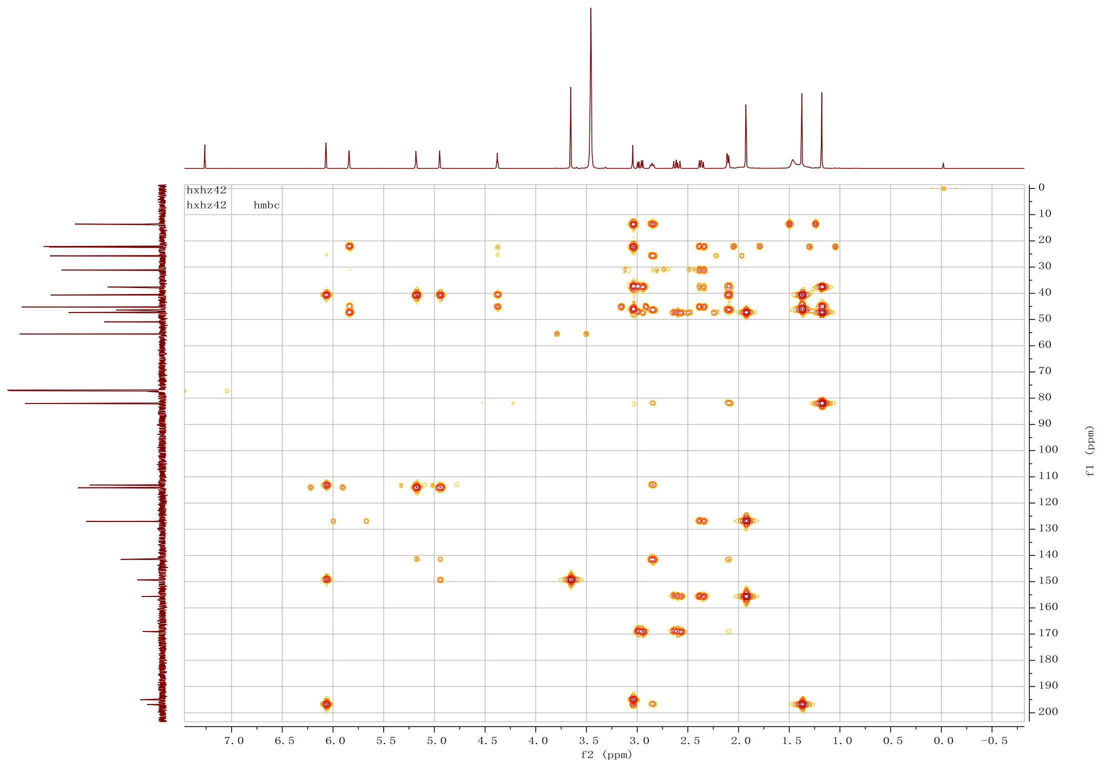

Figure S35. HMBC spectrum of Perforalactone K (6) in $CDCl_3$.

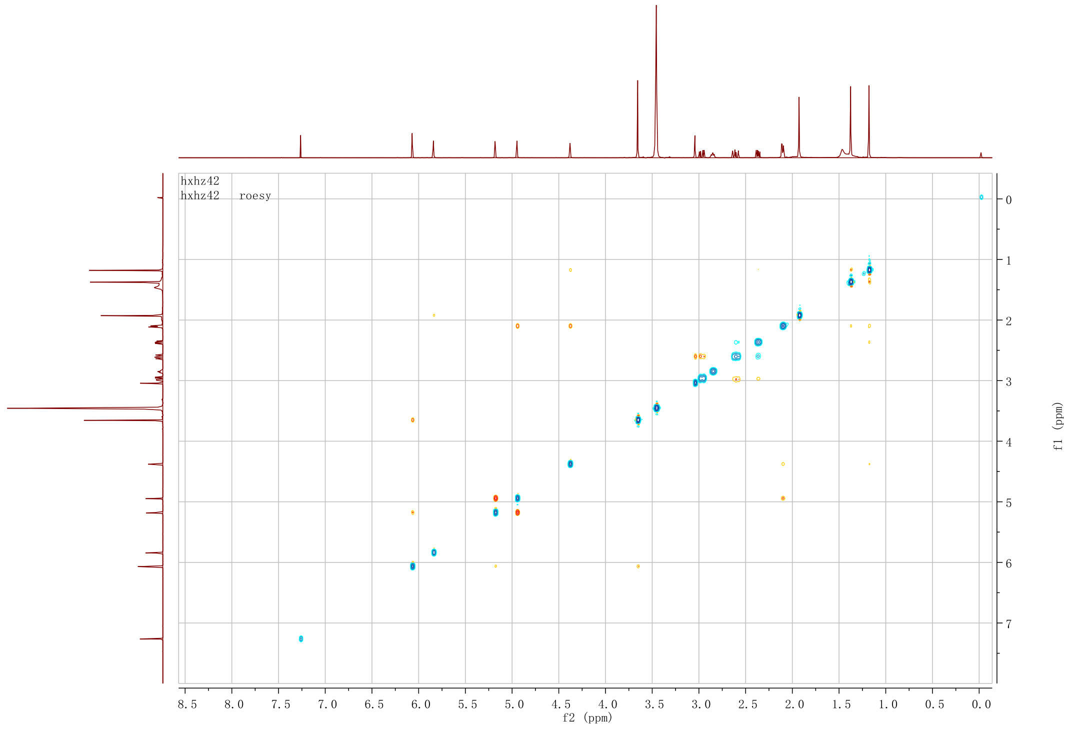

Figure S36. ROESY spectrum of Perforalactone K (6) in $CDCl_3$.

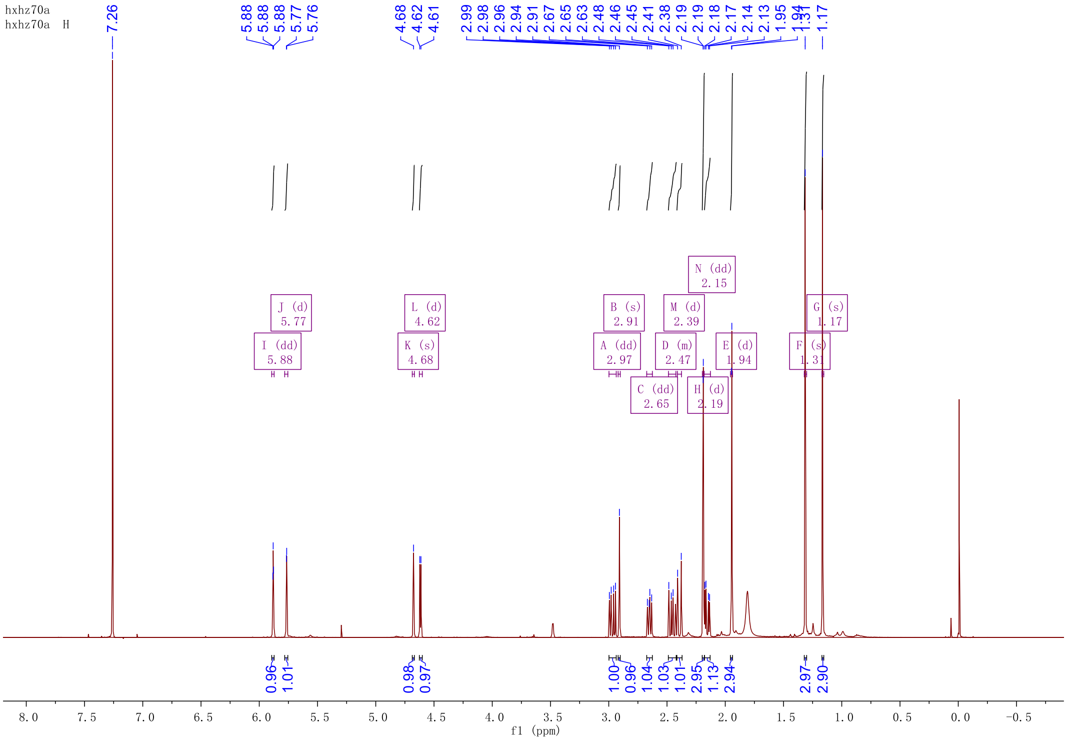

Figure S37. ^{1}H NMR spectrum of Perforalactone L (7) in CDCl₃.

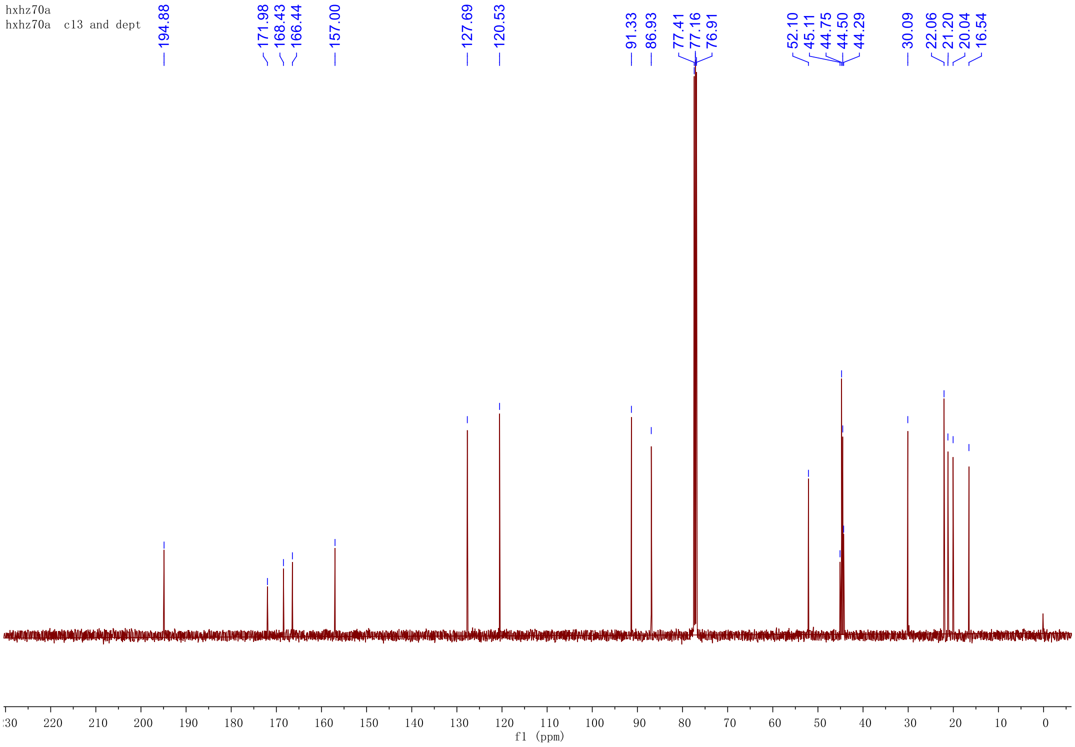

Figure S38. ^{13}C NMR spectrum of Perforalactone L (7) in CDCl₃.

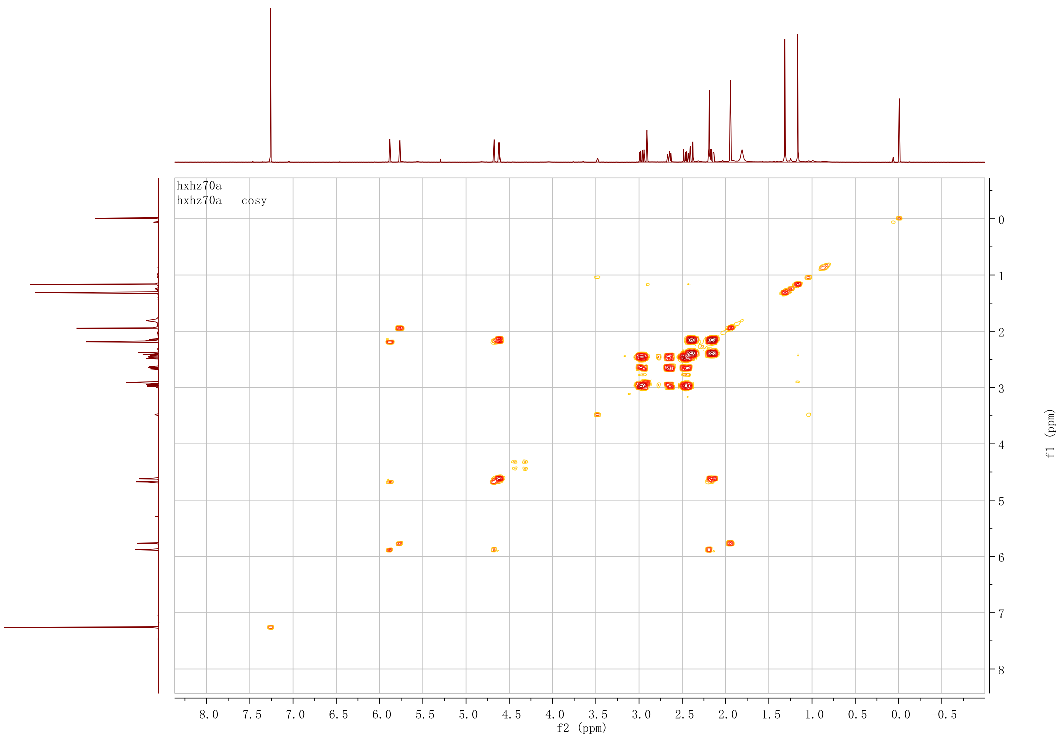

Figure S39. ^{1}H-^{1}H COSY spectrum of Perforalactone L (7) in $CDCl_3$.

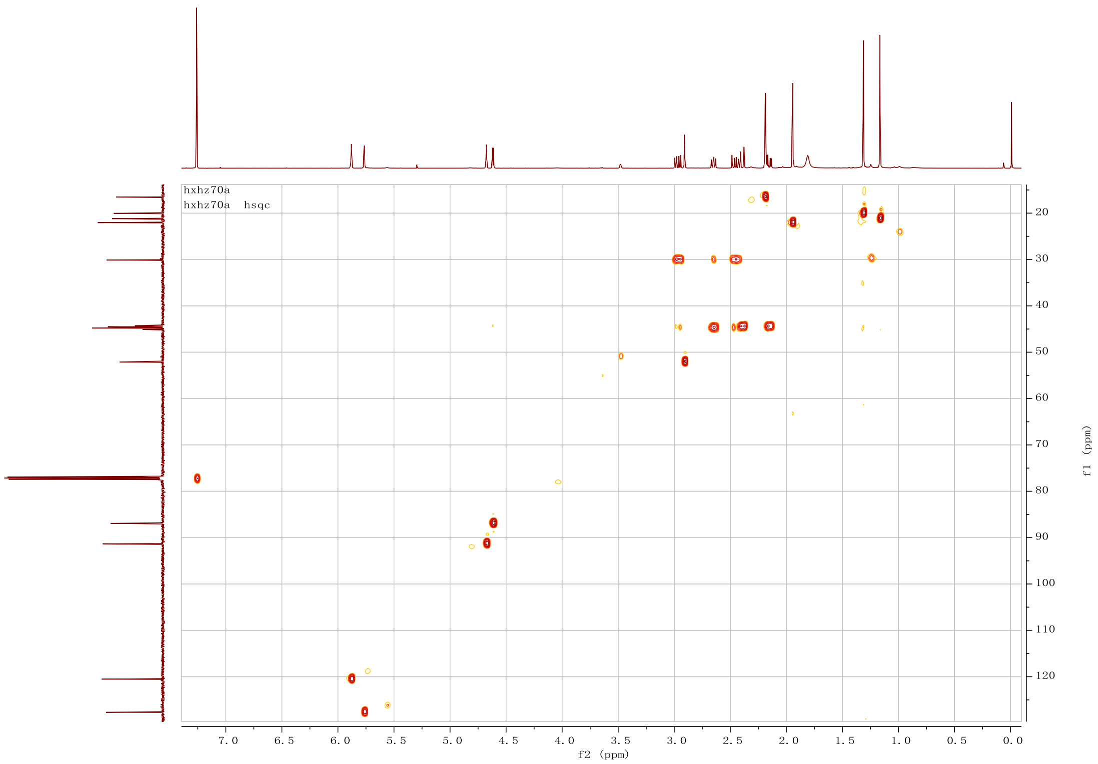

Figure S40. HSQC spectrum of Perforalactone L (7) in $CDCl_3$.

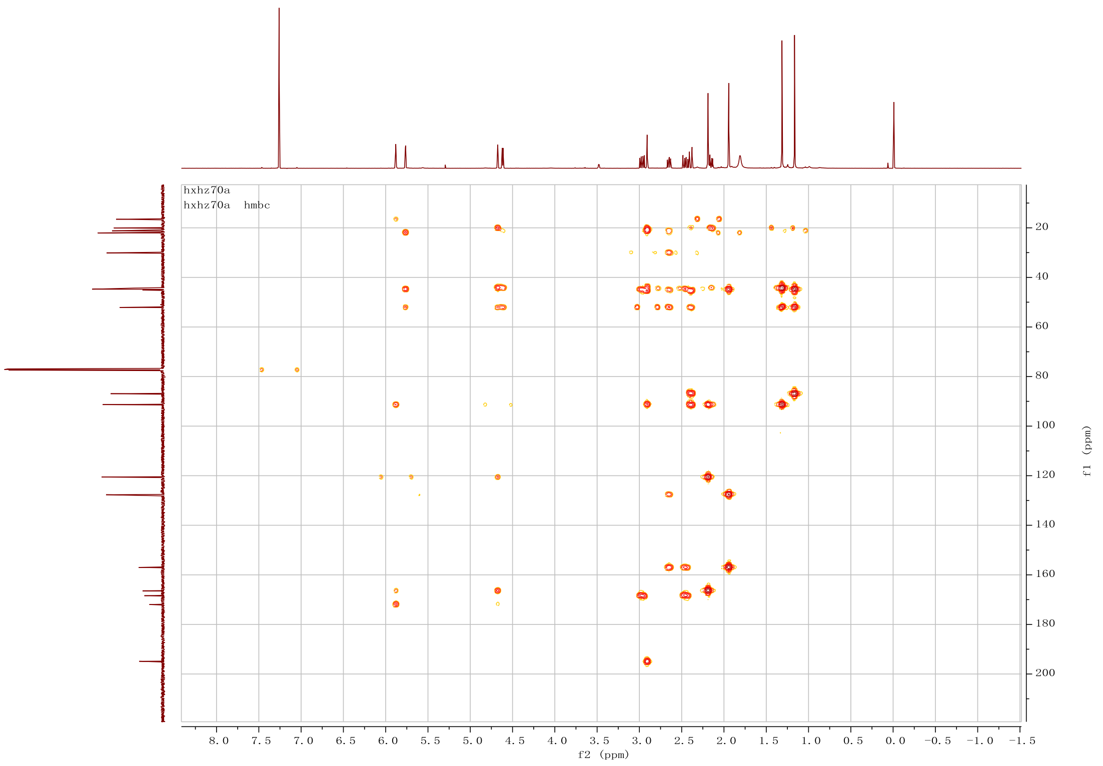

Figure S41. HMBC spectrum of Perforalactone L (7) in $CDCl_3$.

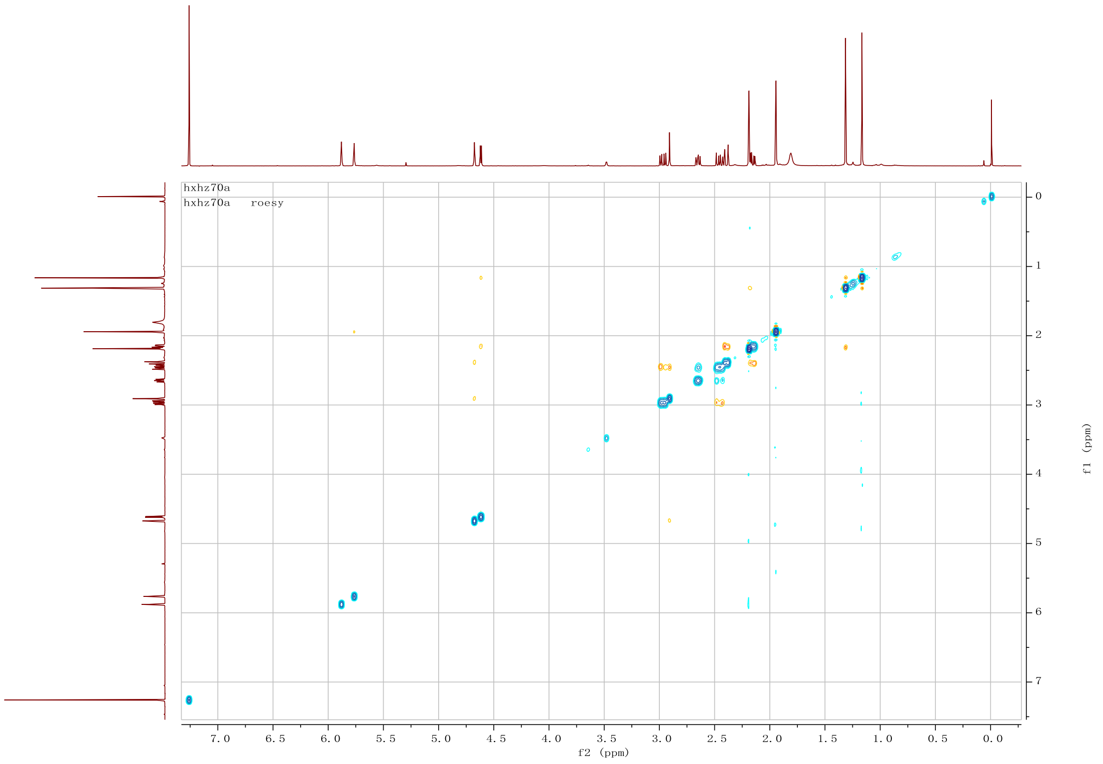

Figure S42. ROESY spectrum of Perforalactone L (7) in $CDCl_3$.
